# Scalable hybrid deep neural networks/polarizable potentials biomolecular simulations including long-range effects†

**DOI:** 10.1039/d2sc04815a

**Published:** 2023-04-04

**Authors:** Théo Jaffrelot Inizan, Thomas Plé, Olivier Adjoua, Pengyu Ren, Hatice Gökcan, Olexandr Isayev, Louis Lagardère, Jean-Philip Piquemal

**Affiliations:** a Sorbonne Université, Laboratoire de Chimie Théorique UMR 7616 CNRS Paris 75005 France jean-philip.piquemal@sorbonne-universite.fr; b Department of Biomedical Engineering, University of Texas at Austin Austin Texas USA; c Department of Chemistry, Carnegie Mellon University Pittsburgh Pennsylvania USA; d Sorbonne Université, Institut Parisien de Chimie Physique et Théorique FR 2622 CNRS Paris France

## Abstract

Deep-HP is a scalable extension of the Tinker-HP multi-GPU molecular dynamics (MD) package enabling the use of Pytorch/TensorFlow Deep Neural Network (DNN) models. Deep-HP increases DNNs' MD capabilities by orders of magnitude offering access to ns simulations for 100k-atom biosystems while offering the possibility of coupling DNNs to any classical (FFs) and many-body polarizable (PFFs) force fields. It allows therefore the introduction of the ANI-2X/AMOEBA hybrid polarizable potential designed for ligand binding studies where solvent–solvent and solvent–solute interactions are computed with the AMOEBA PFF while solute–solute ones are computed by the ANI-2X DNN. ANI-2X/AMOEBA explicitly includes AMOEBA's physical long-range interactions *via* an efficient Particle Mesh Ewald implementation while preserving ANI-2X's solute short-range quantum mechanical accuracy. The DNN/PFF partition can be user-defined allowing for hybrid simulations to include key ingredients of biosimulation such as polarizable solvents, polarizable counter ions, *etc.*… ANI-2X/AMOEBA is accelerated using a multiple-timestep strategy focusing on the model's contributions to low-frequency modes of nuclear forces. It primarily evaluates AMOEBA forces while including ANI-2X ones only *via* correction-steps resulting in an order of magnitude acceleration over standard Velocity Verlet integration. Simulating more than 10 μs, we compute charged/uncharged ligand solvation free energies in 4 solvents, and absolute binding free energies of host–guest complexes from SAMPL challenges. ANI-2X/AMOEBA average errors are discussed in terms of statistical uncertainty and appear in the range of chemical accuracy compared to experiment. The availability of the Deep-HP computational platform opens the path towards large-scale hybrid DNN simulations, at force-field cost, in biophysics and drug discovery.

## Abstract 2

Deep-HP is a scalable extension of the Tinker-HP multi-GPU molecular dynamics (MD) package enabling the use of Pytorch/TensorFlow Deep Neural Network (DNN) models.

## Graphical abstract

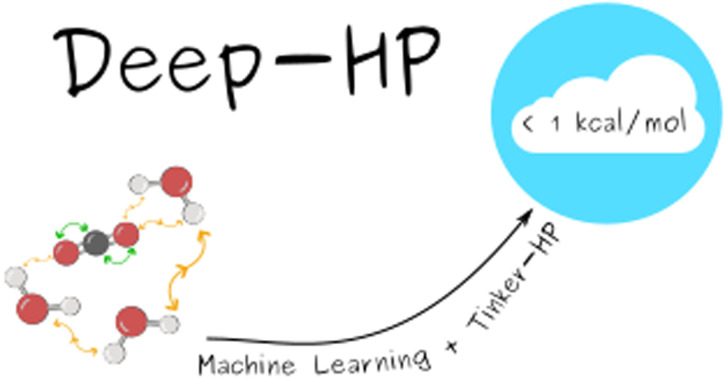

## 1 Introduction

Understanding the dynamics of biological systems is of prime importance in structural biology and drug discovery. Over the last 50 years, coupled to force fields (FFs), molecular dynamics (MD) simulations have proven to be an essential theoretical tool to predict the long-timescale behaviour of proteins in complex environments. In recent years, deep learning technologies have also progressed and showed some potential to accelerate drug discovery. For example, DeepMind developed the Alphafold^2^ (ref. 1) model that is able to predict over 200 million protein structures. Proteins' properties could, however, drastically change during a molecular dynamics simulation. For instance, the protein–water interface can drive fluctuations of catalytic cavities and thus change drug inhibition. MD is therefore the prominent approach to go beyond simple structures in order to predict the complete protein conformational space.^2–4^ Due to the biological system sizes and biological simulation timescales, pure quantum chemistry models cannot be used for simulations and are replaced by empirical FFs, which are presently commonly used to model chemical interactions.

FFs model the total energy as a sum over intra and intermolecular energy terms. The treatment of the latter leads to two classes of FFs: classical and polarizable. In classical FFs, the intermolecular interactions are modeled by Lennard-Jones potential and Coulomb potential which make them computationally efficient enabling modern software to tackle long timescale simulation of complex systems.^5–8^ While offering reasonable precision thanks to careful parametrization,^9,10^ classical FFs lack an accurate description of polarization and to a larger extent of many-body physical effects.^11,12^ These quantities can play a crucial role in solvation^2,3^ and in the stability of secondary and quaternary structures of proteins.^12^ The development of polarizable FFs (PFFs) has opened new routes able to explicitly include many-body effects.^13,14^ Their computational cost has long hindered their use but with the rise of High Performance Computing (HPC)^15,16^ and the increasing performance of computational devices such as GPUs, million-atom PFF simulations are now possible.^17^

At this stage, Machine Learning (ML) schemes also have the potential to offer a new paradigm for boosting MD simulations and to play their role in the development of FFs. ML potentials (MLPs) also avoid solving the Schrödinger equation at each time-step of the simulation by providing a mathematical direct relationship between the atomic positions and the potential energy. In recent years, MLPs have been an active field of research which led to the emergence of different frameworks such as high-dimensional deep neural network potentials (HDNNPs), Gaussian approximation potentials,^18^ moment tensor potentials, spectral neighbor analysis potentials,^19^ atomic cluster expansion, graph networks, kernel ridge regression methods,^20^ gradient-domain machine learning^21–24^ and support vector machines.^25^ MLP nonlinear functional forms are very general and highly flexible, allowing for a very accurate representation of electronic structure computation reference data. The input of an MLP is usually hand-crafted real valued functions of the coordinates that preserve some symmetries and uniquely defined atomic environments. In practice, the choice of this descriptor is central to designing an accurate MLP. A variety of physics-based descriptors have been developed such as the smooth overlap of atomic positions,^26^ the spectrum of approximated Hamiltonian matrix representations,^27^ the Coulomb matrix and the atom-centered symmetry functions.^28,29^ The latter, introduced by Behler and Parinello in 2007, is still the most popular descriptor used for HDNNP and has been employed in numerous studies.^28,30^ It describes the atomic environment of a given central atom inside a cutoff radius *R*_c_ by the use of radial and angular functions. Some modifications of the initial symmetry functions have been done since, aiming to reduce the number of symmetry functions that exhibit quadratic growth with the number of elements or improve the probing of the atomic environment.^31^ However, even if such descriptors have considerably improved the transferability and the scalability of HDNNPs, they are often used to only study small chemical systems that remain far away from the needs of biological modeling. They have nevertheless already been shown to be useful to create buffer region neural network in QM/MM (Quantum Mechanics/Molecular Mechanics) simulations to minimize overpolarization artifacts of the QM region due to classical MM.^32^ Another issue has been the lack of efficient MLP multi-GPU infrastructure software inside an already existing molecular dynamics package. In the last couple of years things started to change and our work is part of this large movement and also aims to address the recent development of the ML-field.^33^ While our work aims to utilize new developments in the ML-field, we also aim to address some of the shortcomings of MLPs. Indeed, the intrinsic architecture of MLP usually constrains them to short-range interactions. Recently, Tsz Wai Ko *et al.* proposed a fourth-generation of HDNNP which is able to capture long-range charge transfer and multiple charge states.^34^ While it demonstrates the power of ML, its computational cost is much higher compared to physics-based PFF long-range models and is not yet able to correctly describe a solute in water.

To address these challenges, we present Deep-HP (HP stands for High-Performance), a multi-GPU MLP platform which is part of the Tinker-HP package and enables the coupling and development of MLPs with state-of-the-art many-body polarizable effects. Tinker-HP uses massive parallelization by means of 3D decomposition which is a particularly well suited strategy for MLPs that are often developed by decomposing the total energy as a sum of atomic energy contributions.^15,17^ The platform theoretical scalability with MLPs is linear and allows scaling up to hundreds/thousands of GPUs on large systems. As the present code shares the Tinker-HP capabilities, it allows for invoking fast physics-based many-body energy contributions. We extensively test Deep-HP scalability and implementation on the ANI model, one of the most accurate MLPs to date for small organic molecules. Finally, in the spirit of polarizable QM/MM embedding simulations,^35–37^ we introduce a hybrid DNN/MM strategy that uses the ANI DNN to model solute–solute interactions and the AMOEBA PFF to evaluate solvent–solute and solvent–solvent interactions. This enables ANI to benefit from AMOEBA's strengths that include an accurate condensed phase flexible water and protein model, and the capability to include counter-ions and long-range/many-body effects. It should increase ANI transferability to a broader range of systems including charged ones. The performance of the model is evaluated by calculating the solvation free energies of various molecules in four organic solvents as well as the binding free energies of 14 challenging host–guest complexes taken from SAMPL blind challenges.

## 2 Method

### 2.1 Potential energy models

#### 2.1.1 The AMOEBA polarizable force field

The total potential energy of the AMOEBA^38,39^ polarizable model is expressed as the sum of bonded and non-bonded energy terms:1
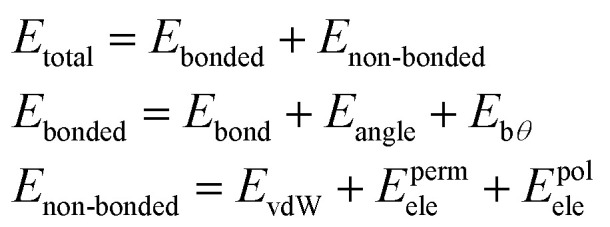


The bonded terms embody MM3-like^40^ anharmonic bond-stretching and angle-bending terms. Regarding the specific case of the polarizable AMOEBA water model, the intramolecular geometry and vibrations are described with a Urey–Bradley approach.^38^

The non-bonded terms include van der Waals interactions and electrostatic contributions from both permanent and induced dipoles (polarization). More precisely, the polarization contribution is computed using an Applequist/Thole model^41^ whereas Halgren's buffered 14–7 pair potential is used to model van der Waals interactions.^42^ Computing the polarization energy requires the resolution of a linear system to get the induced dipoles, which is made through the use of iterative solvers such as a preconditioned conjugated gradient that is the one used in this paper (with a 10^−5^ tolerance).^17^

To model the electrostatic interactions, AMOEBA relies on point atomic multipoles truncated at the quadrupole level. More details about the functional form and parametrization of AMOEBA can be found in ref. ^43^ Electrostatics and many-body polarization long-range interactions are fully included through the use of the Smooth Particle Mesh Ewald approach^44,45^ that allows for efficient simulations in periodic boundary conditions with *n*(log(*n*)) scaling. Besides water,^38^ AMOEBA is a general force field available for the biomolecular simulations of many solvents,^46^ ions,^47,48^ proteins^49^ and nucleic acids.^50^

#### 2.1.2 Neural network potentials

Feed-forward neural network (FFNN) is a machine learning model that uses as building blocks connected layers of nodes (*i.e.*, neurons) each associated with their weights and bias. The output of each neuron is computed through a function of the output of the previous layer. Each weight is the strength associated with a specific node connection and they are updated during the training process. The depth (*i.e.*, number of layers) of the FFNN is related to its flexibility and the complexity of the training dataset. Through careful optimization of hyperparameters, weights, biases and architecture, the FFNN can learn high dimensional non-linear functions such as potential energy surfaces. For HDNNP, the FFNN maps molecular structures to potential energy. The original HDNNP, introduced by Behler and Parrinello, expresses the total energy of a system *E*_T_ as a sum of atomic contributions *E*_*i*_.2
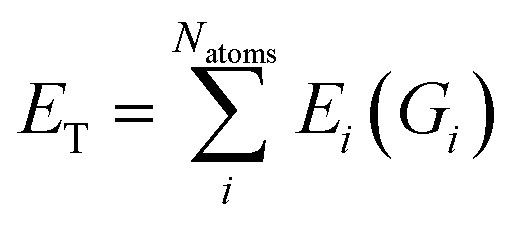
where *G*_*i*_ is the atomic environment vector (AEV) of atom *i*. Based on the assumption of locality, each atom *i* is associated with an AEV which probes specific radial and angular chemical regions. Each *G*_*i*_ is then used as the input into a single HDNNP. The construction of AEVs for each atom in the system enables the use of models for large systems even though they are trained on small molecules. Moreover, this summation has the advantage that it scales linearly with respect to the number of atoms. This atomic decomposition scheme has notably accelerated the development of HDNNP with increasingly complex architecture and AEV schemes.

#### 2.1.3 ANI models

Smith *et al.* developed ANI, a model that uses a modified version of the Behler–Parinello symmetry functions.^31,51^ Symmetry functions are building blocks of the so-called AEV, *G*_*i*_ = {*G*^*X*^_1_,…,G^X^_M_}, which aims to probe the angular and radial local environment of a central atom *i* with atomic number *X*. The locality approximation is achieved by using a differentiable cutoff function:3
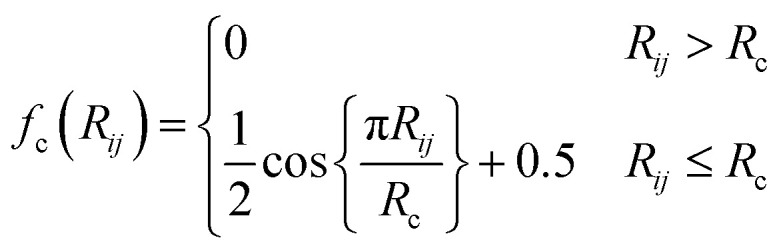
where *R*_*ij*_ is the distance between the central atom *i* and a neighbor *j*, and *R*_c_ a cutoff radius, here fixed to 5.2 Å. To probe the neighboring environment of the central atom inside the cutoff sphere, the AEV is divided into two types of symmetry functions: radial and angular.

The commonly used radial function is a sum of products of Gaussian and cutoff functions as introduced by Behler–Parinello:4
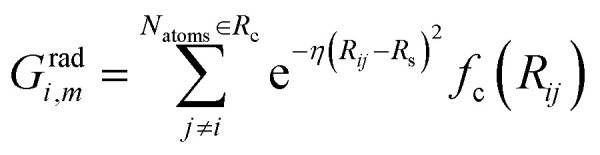


The index *m* is associated with a set of parameters {*η*, *R*_s_}, where *R*_s_ is the distance from the central atom for which the center of the Gaussian is shifted and *η* is the spatial extension of the Gaussian.

The radial symmetry functions are not sufficient to distinguish between chemical environments, *e.g.*, if the neighboring atoms are all at the same distance from atom *i*. This is solved by using angular symmetry functions,5
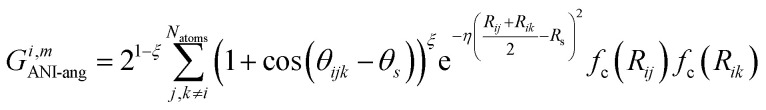
where *θ*_*ijk*_ is the angle between the central atom *i* and neighbors *j* and *k*, *θ*_s_ is used to center the maxima of the cosine and *ξ* changes the width of the peak. To differentiate between atom species, ANI supplied a radial part for each atomic number and an angular part for each corresponding pair inside the cutoff sphere *R*_c_. Thus, for *N* atom species, the AEV has *N* radial and 
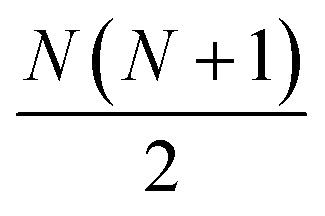
 angular sub-AEVs.

The first ANI potential, ANI-1X,^52,53^ has been developed for simulating organic molecules containing H, C, N, and O chemical elements. The recent extension to ANI, ANI-2X,^54^ has been trained to three additional chemical elements (S, F, and Cl). This model extends the capabilities of ANI towards more diverse chemical structures such as proteins that often contain sulfur and chlorine atoms.^54^

As ANI is mainly designed to study the dynamics of small-to medium-size organic molecules, it had not been initially coupled to a massively parallel infrastructure. In contrast, another popular MLP, introduced by Car and collaborators,^55,56^ DeePMD has been pushed towards large scale simulations of millions of atoms but has been trained on some specific systems, limiting its transferability.

#### 2.1.4 DeePMD models

The specificity of DeePMD compared to other MLPs is that it does not use hand-crafted symmetry functions to get the atomic environment.^55,56^

For an atom *i*, its *j* neighbors within a cutoff radius are first sorted according to their chemical species and their inverse distances to the central atom.

The central atom is then associated with its local frame (*e*_*x*_, *e*_*y*_, *e*_*z*_) and the local coordinates of its neighbors are denoted as *x*_*ij*_, *y*_*ij*_, *z*_*ij*_. The local environment of atom *i* {*D*_*ij*_} is then defined as:6
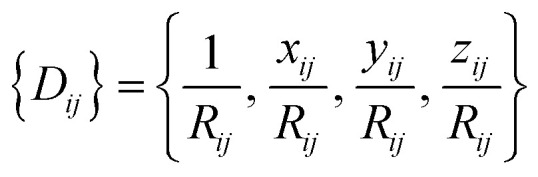


{*D*_*ij*_} is then used as input for an FFNN to predict the atomic energy *E*_*i*_.

DeePMD has been recently pushed in order to simulate tens of millions atoms for water and copper using a highly optimized GPU code on the Summit supercomputer^33^ but it would hugely benefit from all the available features of Tinker-HP in order to run large scale biological simulations.

#### 2.1.5 Hybrid model: neural network solutes in AMOEBA polarizable solvent/protein

Hybrid DNN/MM simulations using classical FFs have been introduced by Lahey and Rowley.^57^ One technical issue with hybrid DNN/FF approaches is that in local MLP models such as ANI and DeePMD, each atom only interacts with its closest neighbors within a relatively small cutoff radius. Therefore, a correct description of long-range interactions is crucial for the simulation of condensed-phase systems, making them particularly challenging for MLP models.^58^ On the other hand, particular attention has been paid during the AMOEBA parametrization to accurately reproduce condensed-phase properties of solvents (and in particular of liquid water). It is then very attractive to combine both models in order to benefit from the best of both worlds getting the small molecule quantum mechanical quality of ANI while maintaining the robustness of AMOEBA for condensed phase simulations. This can be achieved by writing the total potential energy of the so-called ANI-2X/AMOEBA hybrid model as

7 *V*_HYB_(*P* ∪ *W*) = *V*_AMOEBA_(*P* ∪ *W*) + *V*_ML_(*P*) − *V*_AMOEBA_(*P*) = *V*_AMOEBA_(*W*) + *V*_AMOEBA_(*P* ∩ *W*) + *V*_ML_(*P*)

where *P* indicates the solute, *W* indicates the solvent, *P* ∩ *W* indicates the solute–solvent interactions and *P* ∪ *W* indicates the total system. The many-body nature of the polarization energy prevents us from directly computing *V*_AMOEBA_(*P* ∩ *W*). To embed the ML potential, we subtract the AMOEBA potential of the isolated solute to the full AMOEBA potential. As indicated in eqn (7), this is essentially equivalent to using AMOEBA for the solvent–solvent and solvent–solute interactions and the ML model for the solute–solute interactions. The atomic environments that are given to the ML potential therefore only comprise atoms from the solute and should be similar to data present in the training set, thus reducing occurrences of extrapolation. This coupling with AMOEBA allows simulation of atom types not available with MLPs and inclusion of counter ions that are crucial in biology. This also enables the use of the accurate AMOEBA water model while benefiting from the automatic inclusion of long-range effects *via* AMOEBA's efficient Particle Mesh Ewald periodic boundary conditions.

### 2.2 Deep-HP: a multi-GPU MLP platform within Tinker-HP

#### 2.2.1 A general machine learning platform

New ML architecture is introduced daily and dedicated machine learning libraries, PyTorch, TensorFlow and Keras, have created a large community of developers and users.^59–61^

Conversely, most of the MD codes (CHARMM, GROMACS, Tinker-HP,…)^7,62^ are often written using compiled languages such as Fortran or C/C++. To allow for the simultaneous execution of both Python-based MLP codes and Tinker-HP we implemented an interface that allows for efficient data exchanges between environments while maintaining Tinker-HP as the master process which, punctually, calls the MLP code. Identified by Tinker-HP as another computational subroutine, the MLP code should be therefore provided as a Python API. We have implemented such functionality using the C Foreign Function Interface (cffi) for Python which allows for efficient API embedding, within a dynamic library to be linked with. Technically, within such a framework we can now call Python frozen codes from C using such cffi embedding features, thus enabling the use of various MLP codes within Tinker-HP.

In that context, the recent GPU-accelerated version of Tinker-HP^17^ offers the opportunity to build an overall very efficient hybrid MD/MLP code as both applications are running on the same GPU platform. To do so, we need to design a Python/C interface in a way that avoids any substantial data transfers between Python and C environments. In practice, the cffi module is not natively designed to interface data structures from device memory: its dictionary can only process host addresses on array datatype or scalar data structures. Based on these constraints, our code would be forced to perform two host-device data transfers in order to communicate through Fortran/C and Python interface. To overcome this issue that would be detrimental to the global performance, we directly send generic memory addresses through the interface as scalar values and use the PyCUDA python module to manually cast these addresses into Tensor type that can actually be used by MLP codes. Fortunately, PyCUDA and PyTorch provide such casting routines. Thus, calling Python codes from Fortran/C with device data among the calling arguments can be done independent of the size of those arguments.

Furthermore, we built the interface of the MLP code in order to keep Tinker-HP model-agnostic. In practice, Tinker-HP provides positions and neighbor lists and gets energies and forces in return. Adding a new MLP to the platform then becomes an easy task, especially if it was developed using the PyTorch or TensorFlow libraries. Moreover, we implemented an API within TorchANI which allows us to save and reconstruct ANI-like models using JSON, YAML and PKL formats. This allows us to directly use models trained with TorchANI with the Deep-HP platform, thus reducing the hassle of transferring a model from the training stage to production simulations.

#### 2.2.2 Massive parallelism within Tinker-HP: scalable neural network simulations

**Fig. 1:**
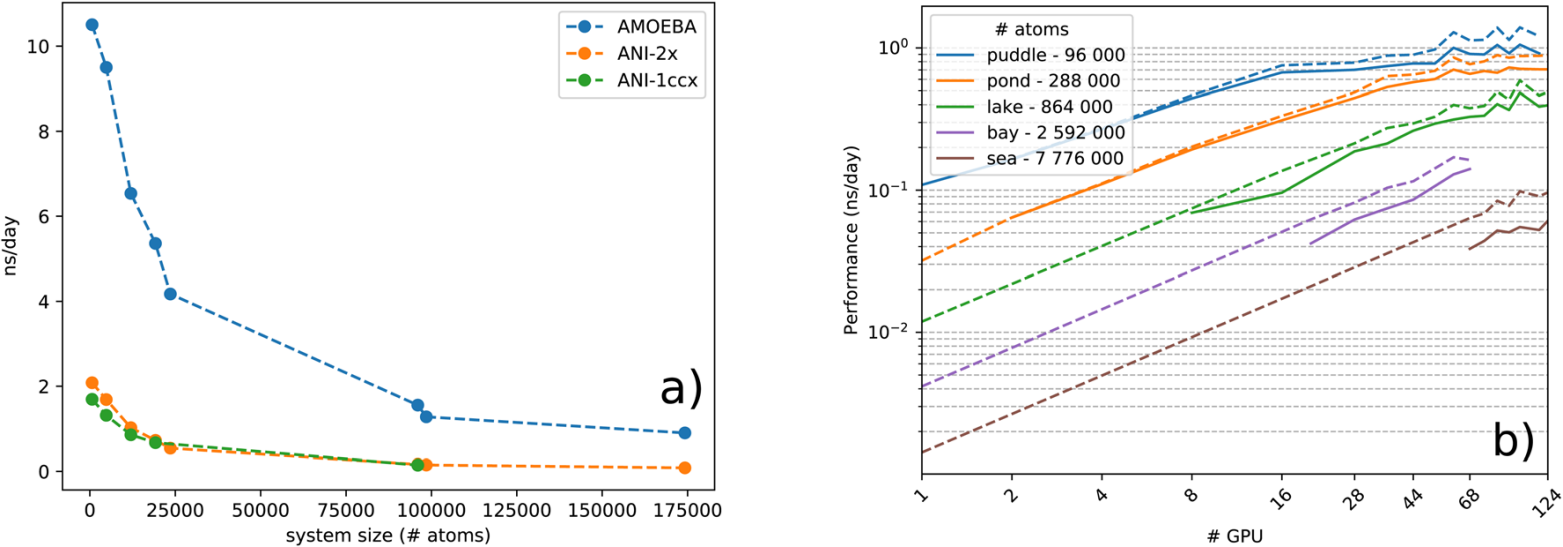
(a) Performance comparison between ANI-1ccx(1NN), ANI-2X(1NN) and AMOEBA models in ns per day, over increasing system size, on a single Nvidia Tesla A100. (b) Strong scaling logarithmic scale plot of the ANI-2X model on benchmark systems. Simulations are performed in the *NVE* ensemble using a Velocity-Verlet integrator 0.2 fs time-step.

Regarding parallelism, Tinker-HP uses a three-dimensional domain decomposition (DD) scheme. The simulation box is decomposed into a certain number of domains matching the exact number of parallel processes at our disposal so that each process – attached or not to a device – is assigned to a unique domain. Then, each process computes partial forces on the local atoms, communicates the partial data to its spatial neighbors, sums the partial forces and integrates the equations of motions for local atoms at each time-step. The DD method is valid and effective under the assumption that all interactions are short-range and the atomic positions do not move much between two time-steps. The same structure has been used during the development of the accelerated multi-GPU version.^17^ Naturally, we wanted to preserve this property with the MLP code interface despite the fact that TorchANI is not designed to run on multiple GPUs. Using the DD method from Tinker-HP, we can isolate the local atoms of a domain and its neighbors and send the information to an MLP code instance through the interface for calculation. We also bypass the implemented neighbor list within TorchANI, and use the one of Tinker-HP. Indeed, we verified that the TorchANI neighbor list algorithm scales as 
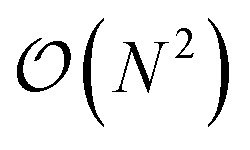
 (*N* being the number of atoms), both in execution time and memory, which limits its applicability to small systems. For instance, a 12 000 atom water box on a Quadro GV100 GPU card supported by 32 GB memory already caused a memory overflow. Because TorchANI requires a pair list of indices as a data structure, we adapted the highly GPU-optimized linked-cell method, thoroughly described in ref. 17. In practice, the list is built by partitioning the box into smaller ones and resorting to an adjacency matrix and a filtering process. Finally, the complexity of the neighbor list generation outperforms the original TorchANI implementation, thus significantly reducing both the computational cost and memory footprint and allowing the handling of much larger systems. For example, systems made of more than 100 000 atoms are now manageable on a single 32 GB GV100 GPU. On top of that, we also noticed a constant memory allocation from Python (especially when running in parallel) which happens to be detrimental to the performance and, on some occasions, can lead to a crash. This issue has been solved by resorting to an upstream bounded buffer reservation whose size is proportional to the number of atoms in the system. In the end, Deep-HP is able to perform simulations of several million atom systems, as illustrated in Fig. 1 where we show the scalability of the platform on water boxes up to 7.7 million atoms using up to 68 V100 GPUs.

### 2.3 Performance and scalability results

#### 2.3.1 Benchmark systems

We use water boxes of increasing size as benchmark systems as well as some solvated proteins.^15,17^ The solvated proteins and their respective number of atoms, in parentheses, are: DHFR protein (23 558), SARS-CoV2 M^pro^ protein (98 500) and COX protein (174 219). For the water boxes: 648 (*i.e.*, small), 4800 (big), 12 000 (huge), 19 200 (globe), 96 000 (puddle), 288 000 (pond), 864 000 (lake), 2 592 000 (bay) and 7 776 000 (sea). After equilibration, we evaluated the performance on short *NVE* MD simulations.

#### 2.3.2 GPU performances

To ensure the performance and portability of our platform, we ran tests on different GPU infrastructures such as Tesla V100 nodes of the Jean-Zay supercomputer, the Irène Joliot Curie ATOS Sequana supercomputer V100 partition or a NVIDIA DGX A100 node. In the rest of the text the default device is the Tesla V100 if not mentioned otherwise. For each system, we performed 2.5 ps MD simulations with a Verlet integrator using a 0.5 fs time-step and averaged the performance over the complete runs. Fig. 1 gathers single GPU device performances.

Before discussing performance results let us introduce three critical concepts: saturation, utilization and peak performance. Saturation represents the ratio of resources used by the algorithm against the actual resources supplied by the GPU. It is closely related to the degree of parallelism expressed within the algorithm and its practical use in the simulation. Given the fact that recent GPUs provide and execute several thousands of threads at the same time to run calculations on numerous computational cores, complete saturation is naturally not achieved for small systems. On the other hand, the device utilization represents the percentage of execution time during which the GPU is active. As the GPU is driven by the CPU, its utilization heavily depends on both the CPU speed and the amount of code actually offloaded to the device. It is essential to rely on asynchronous computation and to develop a device-resident application in order to achieve a complete GPU utilization over time. Finally, peak performance (PP) describes how an algorithm asymptotically harnesses the computational power of the device on which it operates. Increasing this metric implies maximization of arithmetic operations over memory. However, one can only assess device peak performance in terms of floating point operations when both saturation and utilization are maximized. With a typical HPC device such as Quadro GV100 which delivers over 15.6 TFlop per s in single precision arithmetic (4 bytes), around 69 arithmetic operations can be performed between two consecutive float transactions from global memory, in order to reach the peak performance. Knowing this, we analyze the GPU peak performance of Deep-HP and Tinker-HP AMOEBA, in both separate and hybrid runs, using the reference GV100 card. Results are depicted in Table 1. We can see the influence of device saturation on peak performance while running pure ML models, from the under-saturated DHFR system to the over-saturated COX one. MLPs manage to achieve excellent peak performance on GPU platforms due to the large amount of calculations induced by the numerous matrix-vector products involved. For AMOEBA, on the other hand, the relatively tiny increase of peak performance for both systems – second column of Table 1 – denotes an excellent saturation and utilization of the device, regardless of the size. The overall peak, however, reaches a lower 10.52%, which is still satisfactory given the complexity of the algorithm involved in the PFF calculation.

**Table 1:** Global peak performance in percentage (%) assessed over a 50 femtoseconds MD trajectory. The Quadro GV100 was chosen to be the reference device

System/model	ANI	AMOEBA	Hybrid
DHFR	19.42	9.08	5.16
COX	28.13	10.52	n/a

To study the complexity of the algorithm, we ran the benchmark systems on a single DGX A100 with two ANI models and compared the performance against the AMOEBA force field (see Fig. 1a). The ANI-1ccx simulations are performed on water boxes ranging from 648 to 96 000 atoms. For ANI-2X we also considered three solvated proteins: DHFR, SARS-CoV2 M^pro^ and COX. Furthermore, for these tests, we performed inference using only one instance from the ensemble of eight neural network predictors of the ANI models. On water boxes, ANI-1ccx is found to be between 2% and 7% faster than ANI-2X due to the model's intrinsic complexities. Fig. 1a shows the performance of both ANI-2X and AMOEBA. In the 648 and 4800 atom systems, AMOEBA is 1.85 and 2.20 times faster than ANI respectively. In the first four water systems the ratio grows as 
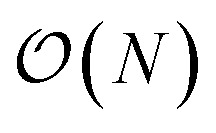
 with respect to the number of atoms *N*, with a Pearson coefficient equal to 0.995. In the protein systems the ratio still grows linearly but with a smaller slope: roughly a factor 2 is preserved.

To further analyze the computational bottleneck of HDNNP models, we evaluated the contribution of each of the model's constituents to the overall execution time (Fig. S1 ESI†). For small systems more than 40% of the cost is due to the gradients and AEV computations. The Tinker-HP neighbor list is less than 5% of the cost, demonstrating the performance of the implementation. For larger systems, the computational cost is largely dominated by the gradient's computation (*i.e.*, more than 50%). Thus, ML potential's computational performances are now mainly limited by back-propagation and not by the environment vector (the latter mainly being the memory bottleneck). Accelerating the gradient's estimation will therefore be of utmost importance for future implementations. Deep-HP also provides a keyword to automatically use mixed precision within PyTorch. The automatic mixed precision is using a combination of half and single precision operations without a severe loss on the model's accuracy.

#### 2.3.3 Multi-GPU performance and scalability of ANI models within tinker-HP

In the following, we assess and discuss the multi-node performance of Deep-HP. The Jean Zay HPE SGI 8600 GPU system holds numerous computing nodes accelerated by 4 interconnected Tesla V100 devices each. Ideally, a parallel algorithm associated with a certain amount of resources (*N* processors for instance), whose load is equally distributed across all resources, will exactly perform *N* times faster. Experimentally, an intermediate step, occupied with communications, affects the performance to a varying degree depending on the size and pattern of these communications in comparison with the amount of calculations. When the number of allocated resources increases, global synchronizations induced by collective communications significantly slow down the parallel execution and, therefore, impact the asymptotic behavior of the strong scalability. Communication patterns and speed are subsequently the principal obstacles to achieve an ideal scaling. In our case, the domain decomposition method coupled with ANI offers an up-bounded communication pattern, which allows the use of several nodes without enduring severe performance loss too quickly, as it is the case with multi-node PFF on GPUs.^17^ As displayed in Fig. 1b, we are able to scale up to 11 nodes (44 devices) for an 864 000 atom water box, before suffering from communication overheads and insufficient load. On the other hand, note that an accurate estimation of the gradients for each atom requires a complete knowledge of its surrounding environment up to a predetermined distance. The current implementation is however not optimal for a large number of processes and the performance starts to cap when half the minimum length of a domain equals the cutoff distance of the atomic environments. This is due to some redundancy between processes for the calculation of AEVs and energies of atoms from neighbouring domains. To illustrate this effect, we made an estimation of the performance in the case of no computational redundancy and plotted it for every test case in dashed lines within Fig. 1b. As anticipated, dealing with this effect can offer a significant 40% boost in the parallel run as is observed for the sea water box. Thus, future implementations should address this issue in order to maximize multi-node performance. The test machines we used were also not optimal and do not provide fast interconnect between nodes. The observed A100 50% boost coupled to improved node interconnections will certainly be extremely beneficial to Deep-HP (we could not get access to a large recent A100 cluster and were limited to a single DGX-A100 node). Nevertheless, the current implementation can already be considered as a game changer for ANI/ANI-2X DNN simulations as the use of several GPUs already provides the capability to produce ns per day molecular dynamics simulations on hundreds of thousands of atom systems (see detailed benchmarks in Table 2).

**Table 2:** Performance of the ANI-2X neural network in Deep-HP in terms of molecular dynamics simulation production (ns per day) for selected water boxes of increasing sizes using Nvidia V100 and A100 GPU cards^a^

Systems (number of atoms)/number of GPU devices	1	4	8	16	28	44	68	84	100	124
**GPU V100**										
Puddle (96 000)	0.11	0.27	0.44	0.67	0.70	0.78	0.91	1.05	1.05	1.05
Pond (288 000)	n/a	0.11	0.19	0.31	0.46	0.57	0.66	0.67	0.71	0.71
Lake (864 000)	n/a	n/a	0.07	0.10	0.19	0.26	0.33	0.40	0.48	0.40
Bay (2 592 000)	n/a	—	n/a	0.04	0.06	0.09	0.14	n/a	n/a	n/a
Sea (7 776 000)	n/a		—	—		n/a	0.04	0.05	0.06	0.06

**GPU A100**										
Puddle (96 000)	0.16	0.41	0.63	n/a			—			n/a
Pond (288 000)	n/a	0.16	0.26	n/a			—			n/a
Lake (864 000)	n/a	n/a	0.11	n/a			—			n/a

**Theoretical performance (V100)**										
Puddle (96 000)	0.11	0.27	0.46	0.75	0.79	0.90	1.14	1.39	1.40	1.40
Pond (288 000)	0.03	0.11	0.20	0.33	0.49	0.65	0.77	0.89	0.88	0.89
Lake (864 000)	0.01	0.02	0.07	0.14	0.21	0.30	0.38	0.49	0.59	0.49
Bay (2 592 000)	0.004	0.007	0.02	0.06	0.08	0.12	0.16	n/a	n/a	n/a
Sea (7 776 000)	0.001	0.003	0.005	0.009	0.02	0.03	0.06	0.08	0.10	0.10

a n/a: not available.

#### 2.3.4 Accelerating hybrid simulations: multi-timestep integrators (RESPA/RESPA1) and reweighting strategies

##### 2.3.4.1 Multi-timestep integrators (RESPA/RESPA1)

As fast as the ANI model can be compared to Density Functional Theory (10^6^ factor speedup), ANI remains far more computationally demanding than polarizable force fields (see the ESI, Tables S1 and S2†) and the stiff intramolecular interactions reproduced by the MLP limits the integration time-step to “*ab initio*” 0.2–0.3 fs values, thus making the study of large proteins on long biological timescales a daunting task. One way to speed up MD is to use larger time steps through multi-time-stepping (MTS) methods thanks to a hybrid model. As discussed in Section 2.5, we decided to introduce the ANI-2X/AMOEBA model, that is, coupling a very accurate MLP for small molecules (ANI) to a PFFS designed to produce accurate condensed phase simulations of solvated proteins (AMOEBA). Typical MTS schemes exploit the separability of the potential energy into a computationally expensive, slowly varying part and a cheap, quickly varying part, and use a specific integration scheme, RESPA,^63^ that allows for less frequent evaluations of the expensive part. In particular, in the context of the AMOEBA PFF, Tinker-HP uses either a bonded/non-bonded splitting or a three-stage separation between bonded, short-range non-bonded and long-range non-bonded interactions^64^ (denoted as RESPA1 in the rest of the text). In both cases, temperature control is made through a BAOAB discretization of a Langevin equation.^65^ In this context, the bonded forces are integrated using a small 0.2–0.3 fs time-step and the outermost time-step can be taken as 2 fs or 6 fs depending on the splitting. These can be further pushed by using Hydrogen Mass Repartitioning (HMR).^64,66^ These integration schemes extend the applicability of PFFs to a longer time-scale reducing the gap with classical FFs, as demonstrated with recent simulations of tens of μs of the SARS-CoV2 M^pro^ protease.^2^

Even though MLPs are much less expensive than *ab initio* calculations, the most common MLPs with feed-forward neural networks remain more computationally demanding than FFs, even polarizable ones (see the ESI, Table S1†). To reduce this gap, towards simulating large biological systems, we combined our hybrid ANI-2X/AMOEBA model to MTS integrators using the RESPA scheme. We assume that AMOEBA is a good approximation of the ML potential for the isolated solute so that their energy difference Δ*V*_ML_(*P*) = *V*_ML_(*P*) − *V*_AMOEBA_(*P*) should produce small forces that can be integrated using a larger time-step. This is done in the same spirit as Liberatore *et al.*^67^ that studied such an integration scheme in the context of accelerating *ab initio* molecular dynamics. We thus associate this difference with the non-bonded part of the AMOEBA model and end up with the following separation:

8 *V*^fast^_HYB_(*P* ∪ *W*) = *V*^bond^_AMOEBA_(*P* ∪ *W*)

9 *V*^slow^_HYB_(*P* ∪ *W*) = Δ*V*_ML_(*P*) + *V*^nonbond^_AMOEBA_(*P* ∪ *W*)

where *V*^fast^_HYB_ is evaluated every inner time-step and *V*^slow^_HYB_ every outer one. In the RESPA1 framework, the potential energy difference Δ*V*_ML_(*P*) is associated with the long-range interactions and evaluated at the outermost time-step.

To assess the accuracy of each integrator we computed the solvation free energy of two solutes with the hybrid model described above: the benzene molecule solvated in a cubic box of 996 water molecules with a 31 Å edge and a water molecule in a cubic box of 3999 other water molecules with a 49 Å edge. For each of these systems and integrators, we computed their solvation free energy by running 21 independent trajectories of 2 ns and 5 ns where the ligand is progressively decoupled from its water environment, first by annihilating its permanent multipoles and polarizabilities and then by scaling the associated van der Waals interactions (while using a softcore). The trajectories were run in the *NPT* ensemble at 300 K and 1 atmosphere using a Berendsen barostat and either a Bussi thermostat^68^ (when Velocity Verlet is used) or a Langevin one for the MTS simulations as mentioned previously. The free energy differences were then computed using the BAR method.^69,70^ Results were compared with a reference Velocity-Verlet integrator using a 0.2 fs time-step. The AMOEBA bonded forces were always evaluated every 0.25 fs. In the case of a bonded/non-bonded split, the non-bonded forces were evaluated either every 1 or 2 fs, and in the case where the non-bonded forces are further split between short-range and long-range ones, the short-range non-bonded forces were evaluated every 2 fs and the long-range ones either every 4 fs or 6 fs. As explained above, the MLP forces are always computed at the outermost time-step.

The accuracy of the results is displayed in Table 3. RESPA1 approaches, despite being operational, appear more sensitive to the system and do not always lead to the desired result in terms of free energies and should be restricted to simple simulation purposes. Therefore, the tighter RESPA (0.25/1 and 0.25/2) integrators are found to be good compromises between accuracy and computational gain. Table 4 shows the speedup of the hybrid model with various MTS setups compared to reference Velocity Verlet ANI-2X/AMOEBA simulation with a 0.2 fs time-step and Velocity Verlet ANI simulations with a 0.2 fs. In practice, speedups are system-dependent, but RESPA techniques always lead to a consequent acceleration compared with the tighter accuracy integration scheme (Verlet) for an ANI solute in a polarizable AMOEBA solvent and compared to pure ANI (Verlet 0.2 fs) simulations. These integrators thus extend the applicability of machine learning-driven molecular dynamics to larger biologically relevant systems and to longer-time-scale simulations. In practice, the resulting performance gain helps to reduce the computational gap between ANI and AMOEBA that is initially about more than a factor 30 (see the ESI, Table S1†).

**Table 3:** Solvation free energy (kcal mol^−1^) comparison for the benzene and water molecules. Comparison between experimental, AMOEBA and hybrid ANI-2X/AMOEBA results using Velocity Verlet, BAOAB-RESPA and BAOAB-RESPA1 integrators. H corresponds to the use of hydrogen mass repartitioning (HMR). Simulations were performed in the *NPT* ensemble with 2 ns and 5 ns (in parentheses) BAR windows, with the BAOAB-RESPA/RESPA1 integrators

	Exp.	AMOEBA	V (0.2)	R (0.25/1)	R (0.25/2)	R1^H^ (0.25/2/4)	R1^H^ (0.25/2/6)
Benzene	−0.87	−0.37	−0.83	−0.97 (−0.90)	−0.87 (−0.88)	−1.69 (−1.69)	−1.60
Water	−6.32	−5.62	−6.33	−6.29 (−6.23)	−6.21 (−6.22)	−6.39 (−6.33)	—

**Table 4:** Relative speedup of hybrid models with RESPA (R) and RESPA1 (R1) integrators calculated with respect

splits	0.2	0.25/1	0.25/2	0.25/2/4	0.25/2/6
Benzene^a^	1.0	4.74	8.42	14.51	18.17
Water^a^	1.0	4.39	8.07	12.58	—
Benzene^b^	1.21	5.74	10.20	17.57	22.00
Water^b^	2.03	8.92	16.40	25.57	—
Integrator-type	V	R	R	R1(HMR)	R1(HMR)

a Hybrid model Velocity-Verlet (V) 0.2 fs time step.

b ANI only with Velocity-Verlet (V) 0.2 fs time step.

##### 2.3.4.2 Accelerating hybrid simulations: an alternative reweighting strategy

Concerning the proposed multi-timestep approach, it is important to note that since we assume that AMOEBA is a good approximation of the ML potential for the isolated solute, the present acceleration strategy is not possible when this condition is not fulfilled. In practice, it could happen in the event of an intramolecular reaction within the DNN solute. Indeed, ANI-2X being a reactive potential, it is sometimes able to produce intramolecular proton transfers in some specific cases, *i.e.*, when donor and acceptor functional groups are present. In contrast, AMOEBA is a non-reactive force field that will always stay in its initial electronic state. Therefore, an intra-ligand chemical reaction would desynchronize the two potentials and therefore stop the simulation. In the rare case of such an event, it is always possible to use a two-step approach and to produce the BAR simulation windows thanks to fast AMOEBA, non-reactive, trajectories. Then one can analyse the AMOEBA snapshots by computing the corresponding ANI-2X/AMOEBA energies to correct the AMOEBA free energy evaluation using a rigorous BAR reweighting^70,71^ (details can be found in the ESI,† see Section 2.2). Such an alternative approach preserves the advantage of speed since the computation of the costly DNN gradients is avoided.

## 3 Results

### 3.1 Solvation free energies

#### 3.1.1 Computational details

To assess further the performance of the ANI-2X/AMOEBA hybrid model, we extended our solvation free energy tests to a variety of small molecules under both aqueous and non-aqueous conditions, as described in ref. 72 and ^73^. The solvents considered, along with their dielectric permittivity values, are as follows: toluene (*ε* = 2.38), acetonitrile (*ε* = 36.64), DMSO (*ε* = 47.24) and water (*ε* = 77.16). Further details regarding the solutes can be found in the ESI.†

We withdrew molecules from the dataset that contained chemical elements not available in ANI-2X, resulting in a total of 38 molecules solvated in water (taken from ref. 43), 20 molecules solvated in toluene, 6 in acetonitrile and 6 in DMSO (taken from Essex *et al.*).^72^ All the systems were prepared following the standard equilibration protocol: after a geometry optimization, they were progressively heated up to 300 K in *NVT* and then equilibrated for 1 ns in the *NPT* ensemble at the same temperature and 1 atmosphere. In all cases, we used the most simple multiple time-step integrator presented above with a 0.25 fs time-step for bonded terms and 1 fs for the outermost one. The Bussi thermostat and the Berendsen barostat were used. The van der Waals interaction cutoff was chosen at 12 Å and the electrostatic interactions were handled with the Smooth Particle Mesh Ewald method^44^ with a 7 Å real space cutoff and default Tinker-HP grid size. We used the same scheme as before to decouple the systems from their environment with 21 independent windows of 2 ns. For solvation free energies in water we also pushed the ANI-2X/AMOEBA simulation windows up to 5 ns. Water as a solvent has been intensively studied as it constitutes a core component driving drug design and as it allows testing for the validity of various computational methods and models.^4,74^ The results are compared with experimental data and with the AMOEBA ones. ANI-2X and AMOEBA standard parametrizations^72,73^ were used.

#### 3.1.2 Results and discussion

**Fig. 2:**
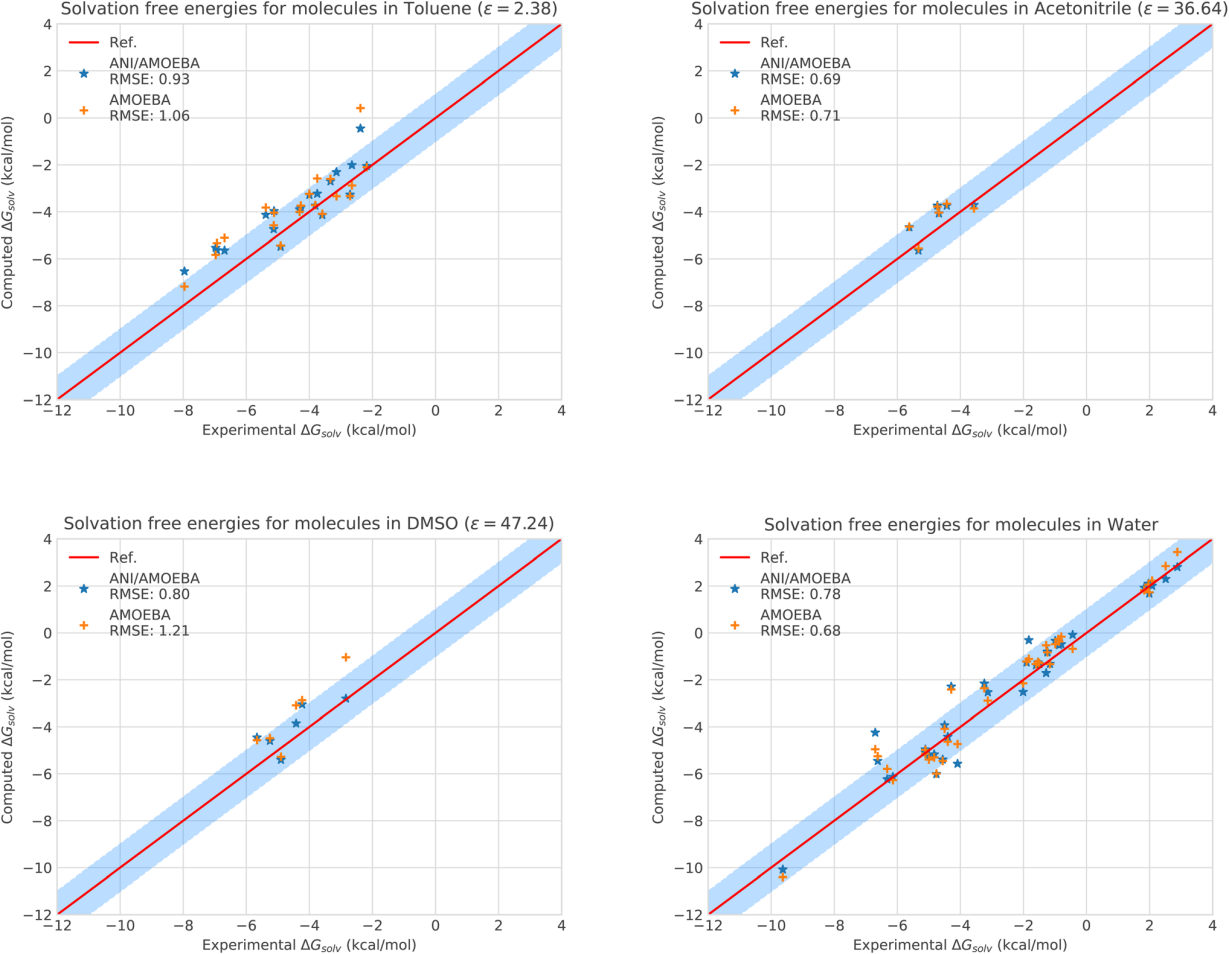
Solvation free energies of molecules in different solvents computed with AMOEBA (orange) from ref. 72 and 73*versus* hybrid model ANI-2X/AMOEBA (blue) and experiment (red). The blue domain corresponds to the so-called chemical accuracy: error of 1 kcal mol^−1^ w.r.t. experiment.

The experimental, AMOEBA and ANI-2X/AMOEBA solvation free energy data are provided in Fig. 2 and Tables S3–S7 of the ESI.† We start with the most challenging solvent, *i.e.* water, which is highly polar and known to be difficult for neural networks. In order to match the recently published AMOEBA Poltype2 (ref. 43) study, we first performed trajectories of 5 ns (instead of 2 ns for other solvents). While we kept most of the poltype2 AMOEBA parameters unchanged, we reparametrized the water, phenol, methylamine and dimethylamine ligands (denoted by R in Table S3†) with the latest version of the Poltype2 software as they were notably performing below the usual AMOEBA standards. Overall, despite the difficult polar solvent, ANI-2X/AMOEBA performs extremely well compared to AMOEBA, exhibiting an RMSE of 0.78 kcal mol^−1^*vs.* 0.68 kcal mol^−1^ for the polarizable force field. This is a very good performance for ANI-2X/AMOEBA since the AMOEBA water model is well-known for its accuracy and capabilities to reproduce numerous water-related experimental data.^38^ To assess the statistical error on solvation free energies, we performed another full run of ANI-2X/AMOEBA (see the ESI, Tables S11 and S12†). The averaged statistical uncertainty amounts for 0.17 kcal mol^−1^ which is consistent with the AMOEBA literature which usually reports errors in the 0.15–0.25 kcal mol^−1^ range for solvation studies.^75,76^ We also investigated the BAR source of error (*via* bootstrapping^76^) which amounts for 0.04 kcal mol^−1^. If a full assessment of statistical errors, *i.e.*, involving multiple simulation replicas is currently out of reach of our computational capabilities due to the use of neural networks, it is nevertheless possible to conclude that ANI-2X/AMOEBA and AMOEBA yield comparable results in water. It is a remarkable result for ANI-2X/AMOEBA that highlights the high accuracy of ANI-2X.

Of course, since water is particularly challenging, we anticipate that ANI-2X would exhibit a gain in accuracy when dealing with apolar solvents. This is clearly the case. For example, for toluene which is a less polar solvent (see Table S5†), the hybrid ANI-2X/AMOEBA results tend to be more accurate than the AMOEBA ones (while staying in the statistical uncertainty), with a respective RMSE of 0.93 kcal mol^−1^*vs.* 1.06 kcal mol^−1^ for AMOEBA. In acetonitrile, ANI-2X/AMOEBA is equivalent to AMOEBA (0.69 kcal mol^−1^*vs.* 0.71 kcal mol^−1^). However, in DMSO, ANI-2X/AMOEBA performs significantly better than AMOEBA, with a respective RMSE of 0.80 kcal mol^−1^*vs.* 1.21 kcal mol^−1^ for AMOEBA. Thus, ANI-2X/AMOEBA and AMOEBA results are within the statistical error for 3 of the studied solvents (including water) while ANI-2X/AMOEBA performs better for DMSO highlighting the high accuracy of ANI-2X. These data confirm the robustness of ANI-2X/AMOEBA in a difficult polar solvent like water once long-range and many-body effects are present. A grasp of its applications will be briefly discussed in the section dedicated to host–guest systems. On the technical point of view, the RESPA acceleration strategy has also been shown to be particularly effective for this solvation study.

In the next section, we go a step further in terms of complexity and report the hybrid model performance on 14 challenging host–guest systems taken from the SAMPL competitions.^77,78^

### 3.2 Host–guest binding free energies: SAMPL challenges

#### 3.2.1 Computational details

This section is dedicated to the measure of the accuracy of the ANI-2X/AMOEBA framework compared to AMOEBA for evaluating host–guest binding free energies. Indeed, AMOEBA is known as one of the most accurate approaches for such studies (see the discussion around the SAMPL challenge^79^) and reaching such accuracy would be a landmark for hybrid neural network simulations. We considered the absolute binding free energy values of 13 guests from the 14 SAMPL4 CB[7]–guest challenge.^78^ We will consider separately the C5 compound that was previously shown^78^ to be a specific outlayer case. We completed the study adding a fourteen complex, the G9 guest taken from the SAMPL6 cucurbit[8]uril host–guest challenge. Free energies were calculated with the hybrid ANI-2X/AMOEBA model as the difference between the free energy of decoupling the ligands within the host and in solution. The optimized structures and parameters for the AMOEBA FF were taken from the literature.^75,78,80,81^ Again, in order to evaluate the impact of the ANI-2X contributions, no AMOEBA specific parametrization has been performed. These ligands are challenging as they are charged, flexible and large, usually leading to difficulties in the prediction of binding free energies.^78^ The same protocol (2 ns windows) as before was used except that the RESPA outer time-step was changed from 1 fs to 2 fs which still gives a satisfactory accuracy, see Table 3. We also provide the free energy values for extended simulations with 5 ns windows in order to explore the accuracy convergence.

#### 3.2.2 Results and discussion

**Fig. 3:**
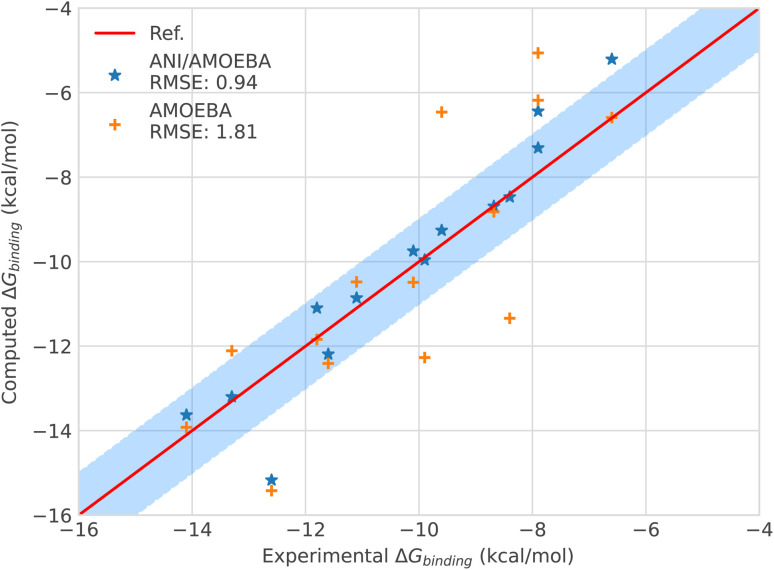
Binding free energies of host–guest systems of the SAMPL4 and SAMPL6 blind challenges with AMOEBA (orange) from ref. 78*versus* hybrid model ANI-2X/AMOEBA (blue) and experimental (red). The blue domain corresponds to the so-called chemical accuracy: error of 1 kcal mol^−1^ w.r.t. experiment.

The binding free energies of the host–guest systems are depicted in Fig. 3 and in Tables S8–S10.† Let's focus first on the accuracy of the ANI-2X/AMOEBA prediction. Overall, the hybrid potential results perform better than the available AMOEBA data reaching an accuracy in the range of chemical accuracy, *i.e.*, 1 kcal mol^−1^ average error w.r.t. experiment. ANI-2X/AMOEBA gives an RMSE of 0.94 kcal mol^−1^*versus* 1.81 kcal mol^−1^ for AMOEBA. It is important to note that in this very challenging testset, all the ligands are charged and encompass a net charge of 1 or 2. As for solvation free energies, the combination of the ANI-2X ligands with the polarizable AMOEBA solvent, host and long-range effects appears to be a powerful tool. Due to computational limitations because of the extensive use of neural networks, we did not resort to extensive statistical error analysis but it is clear that despite the fact that binding free energy uncertainties are usually roughly twice larger than those obtained for solvation studies, ANI-2X/AMOEBA results exhibit a significant improvement over AMOEBA (see a detailed discussion about the statistical uncertainties that one could expect for such studies in ref. 75 and ^76^). If we go more in detail, compound by compound, ANI-2X/AMOEBA exhibits a larger error than AMOEBA for only the C13, C8 and C3 guest ligands. For C13, predictions are both within 0.5 kcal mol^−1^ from experiment. For C8, ANI-2X/AMOEBA also stays within 1 kcal mol^−1^ of error (0.7 kcal mol^−1^). C8 has been shown to be associated with high enthalpy changes throughout binding^78^ and such a change can be traced back to some gains in terms of H-bond interactions from the solution to the host–guest complex. It suggests that improvements of ANI-2X towards improved H-bond treatment could be beneficial. This is consistent with our findings on the solvation free energies where AMOEBA performs slightly better than ANI-2X. Concerning C3, the case is more complex and we review our results below in the same section in link with the discussion on integrators' performances. Only two compound predictions did not reach chemical accuracy: C9 and C10. However, in these cases, the initial AMOEBA error is improved (divided by 2 for C10) using ANI-2X/AMOEBA confirming the higher accuracy of the hybrid model. This result could be associated with slow sampling convergence as noticed by Ren *et al.*^78^ It is worth reporting that in the case of the last compound, *i.e.* the SAMPL6 host–guest system, the ANI-2X/AMOEBA results almost exactly match the experimental results (see the ESI, Table S8†). Finally, we also present in the ESI (Table S10†), the results for the C5 compound that was removed from the testset. These results confirm the initial assessment by Ren *et al.*^78^ and would require further investigation (protonation states, binding modes, sampling time *etc.*…) going beyond the scope of the present work.

Looking in detail at the free energy acceleration strategy, we were overall able to use a RESPA approach on 12 of the 15 (14 + C5) tested ligands. The integrator was not stable enough for the C2, C3 and C4 compounds (see Fig. 3 and ESI, Table S9†). This is due to different reasons. First, C2 and C4 exhibited notably higher differences between the ANI-2X and AMOEBA potentials compared to other ligands. This can be easily understood when considering that C2 and C4 are actually associated with the two largest AMOEBA dataset deviations from the experimental reference values (errors of 3.14 and 2.94 kcal mol^−1^, for C2 and C4 respectively). Since our initial choice was to not perform any specific AMOEBA re-parametrization or ANI-2X dataset modification, the strategy required to either use a tighter, but computationally inefficient Verlet/0.2 fs integration or to perform an ANI-2X/AMOEBA BAR reweighting of a non-reactive AMOEBA set of trajectories, as discussed at the end of Section 2.3.4. Due to the computational constraints, we chose the reweighting strategy that benefits from the efficiency of Tinker-HP to generate AMOEBA trajectories. Table S9 (ESI†) displays the ANI-2X/AMOEBA results obtained for C2 and C4. They are found to be in very good agreement with experiment with errors of 0.34 and 0.07 kcal mol^−1^ respectively. Again, the hybrid potential notably outperforms AMOEBA in these cases as ANI-2X clearly helps to improve the accuracy for these two compounds. For the last ligand, C3, the nature of the problem appeared to be very different as the AMOEBA free energy prediction was almost perfect compared to experiment. In fact, we do not have a parametrization issue here and C3 represents the only case where a reactivity event occurred within our simulations. Indeed, when binding to the host, the C3 ligand adopts a cyclic conformation where its terminal OH and NH_3_ groups strongly interact. This is well captured by AMOEBA. Due to its reactive nature, the ANI-2X potential is able to produce MD trajectories that include proton transfers between the groups suggesting that, for ANI-2X, the compound is actually a mix of two electronic states. As discussed in Section 2.3.4, this situation is simply incompatible with a hybrid RESPA strategy. Again, we performed an ANI-2X/AMOEBA BAR reweighting computation using the well-defined initial AMOEBA electronic state to produce non-reactive classical trajectories. This led to a result apparently less in line with experiment than the AMOEBA one (1.76 kcal mol^−1^*vs.* 0.01 kcal mol^−1^ for AMOEBA) which was anticipated as ANI-2X tends to disfavor the initial state. A solution would be to compute all possible states explored by ANI-2X/AMOEBA. Indeed, many things remain to be solved in the modeling of the SAMPL4 dataset. For example, in the SAMPL4 challenge overview, Muddana *et al.*^81^ reviewed the experimental conditions and concluded that it could be important to take into account the salt conditions and to go beyond the simple box neutralization. Indeed, in the event of a proton transfer, a new ionic species being created, it would be interesting to study its interaction with different solutions of increasing ionic strength, especially in our case where the full simulation includes polarization effects. We have not done it at this stage as it would require a large number of additional simulations and we decided to retain the present C3 free energy prediction that could probably be improved in a forthcoming study. In any case, with C3, ANI-2X brings additional interpretative insights on the nature of the ligand. In the near future, it will also be interesting to investigate further the reactivity capabilities of the ANI-2X/AMOEBA approach. Finally, it is worth noting that C3 is the weakest binder of the series. ANI-2X/AMOEBA still predicts it as such in terms of the relative free energy of binding compared to the other compounds.

Overall, the hybrid ANI-2X/AMOEBA model results are in good agreement with experimental results, reaching, as for the solvation free energy studies, an accuracy in the range of chemical accuracy (average error of 0.94 kcal mol^−1^*vs.* experiment on the dataset) and dividing the initial AMOEBA error by 2. ANI-2X/AMOEBA can accurately predict binding free energies of flexible charged systems and the simulations clearly benefit from the addition of ANI-2X. Finally, in contrast with the results obtained by Lahey and Rowley^57^ that showed the difficulties of the ANI-2X potential for modeling charged systems within a hybrid embedding approach with non-polarizable force fields, we observed accurate results even for charged systems. This is due to a combination of factors linked to many-body and long-range effects and to solvation. Indeed, in the ANI-2X/AMOEBA framework, the charged ligands are embedded in a flexible polarizable solvent that can adapt its dipolar moment to its micro-environment net charges (see ref. 3 and 82 for discussions), providing extra flexibility for the hybrid polarizable embedding approach. For example, the hybrid approach yields good results for nitro-methane, which is globally neutral but still bears two charged groups.

## 4 Conclusion and perspectives

We first introduced Deep-HP, a novel massively parallel multi-GPU neural network platform which is a new component of the Tinker-HP molecular dynamics package. Deep-HP allows users to import their favorite Pytorch/TensorFlow Deep Neural Network models within Tinker-HP. While Deep-HP enables the simulation of millions of atoms thanks to its MPI/domain decomposition setup, it introduces the possibility of reaching ns routine production simulation for hundreds of thousands of atom biosystems with advanced neural network models such as ANI-2X. The platform capabilities have been demonstrated by simulating large biologically relevant systems on up to 124 GPUs with ANI-2X.

Since the platform allows the coupling of state-of-the-art polarizable force fields with any ML potential, we developed a new hybrid deep neural networks/polarizable potential that uses the ANI-2X ML potential for the solute–solute interactions and the AMOEBA polarizable force field for the rest. The development of the hybrid potential was motivated by the capability of AMOEBA to accurately model water–solute and water–water interactions, whereas a neural network such as ANI is better able to capture complex intramolecular interactions at an accuracy approaching the CCSDT(T) gold standard of computational chemistry.^54^

We extended our hybrid model computational capabilities by designing RESPA-like multi-timestep integrators that can speed up simulations up to more than an order of magnitude with respect to Velocity Verlet 0.2 fs. In that context, the relative speedup of AMOEBA compared to the hybrid ANI-2X/AMOEBA dropped from 40 to 2. The hybrid approach offers the inclusion of physically motivated long-range effects (electrostatics and many-body polarization) and the capability to perform efficient Particle Mesh Ewald periodic boundary condition simulations including polarizable counter ions. It also allows us to benefit from the capability of the ANI-2X neural network to accurately describe the ligand potential energy surface leading to high-resolution exploration of its conformational space through the hybrid model MD simulation. The combination of these approaches allows us to treat any type of ligands, including charged ones and opens the door to routine long timescale simulations using NNPs/PFFs up to million-atom biological systems, offering considerable speedup compared to traditional ligand binding QM/MM simulations.

Our hybrid model accuracy was first assessed on solvation free energies of 70 molecules, with a large panel of different functional groups including charged ones, within three non-aqueous solvents and water. The hybrid model is shown to perform well, reaching similar or better accuracy compared to the AMOEBA polarizable force field. Such results open a path towards the simulation of complex biological processes with neural networks for which the environment polarizability is important.^3,4,82^ We then reported the performance of our hybrid model on the binding free energies of 14 host–guest challenging systems taken from the SAMPL host–guest binding competitions. Although most of the ligands are charged, our hybrid model is able to reach performances superior to those of AMOEBA despite the complex chemical environments. Overall, ANI-2X/AMOEBA is shown to reach an accuracy in the range of the chemical accuracy (average errors < 1 kcal mol^−1^ w.r.t. experiment) on the testsets for both solvation and absolute binding free energies. Further work is required to assess the statistical uncertainties linked to such hybrid simulations but the advances in software and HPC will certainly enable such an assessment in the incoming years. Of course, it is important to note that, in some cases, AMOEBA alone is able to reach sub-kcal mol^−1^ accuracy (see for example the SAMPL 8 results).^79^ However, it is not always the case (see SAMPL 6 and 7 results)^75,76^ and seeing a hybrid neural network technology reaching such an accuracy limit is clearly a new step forward.

ANI-2X also provides new features such as the possibility to detect chemical modifications of the ligand thanks to the neural network reactive nature. As the model improves, it could be an important asset for such simulations. As discussed, an accurate AMOEBA parametrization is important and it will be interesting to systematically better converge the level of parametrization of AMOEBA and ANI-2X ligands in order to benefit from maximal multi-timestep acceleration. This should be easily achievable thanks to the recent improvements of the Poltype2 AMOEBA automatic parametrization framework.^43^ In this line, adaptive-timestep alternatives to multi-timestepping using Velocity Jumps^83^ would also be beneficial and are under investigation. These reactivity events also led us to introduce an accurate reweighting strategy. Since it is computationally efficient and avoids the costly computation of DNN gradients, it may become one of the strategies for free energy predictions. Further work will analyse the multiple possibilities of neural network reweighting setups in order to assess their computational efficiency.

Overall, the Deep-HP platform, which takes advantage of state-of-the-art Tinker-HP GPU code, was able to produce within a few days more than 10 μs of hybrid NNPS/PFFs molecular dynamics simulations which is, to our knowledge, the longest MD biomolecular study encompassing neural networks performed to date. Such performances should continue to improve thanks to further Deep-HP optimizations, TorchANI updates and GPU hardware evolutions. Deep-HP will enable the implementation of the next generation of improved MLPs^84–86^ and has been designed to be a place for their further development. It will include direct neural network coupling with physics-driven contributions going beyond multipolar electrostatics and polarization through the inclusion of many-body dispersion models.^87,88^ As Deep-HP's purpose is to push a trained ML/hybrid model towards large scale production simulations, we expect extensions of the present simulation capabilities to other class of systems towards materials and catalysis applications. Overall, Deep-HP allows the present ANI-2X/AMOEBA hybrid model to go a step further towards one of the grails of computation chemistry which is the unification within a reactive molecular dynamics many-body interaction potential of the short-range quantum mechanical accuracy and of long-range classical effects, at force field computational cost.

## Data availability

Deep-HP is part of the Tinker-HP package which is freely accessible to Academics *via* GitHub: https://github.com/TinkerTools/tinker-hp. We are also providing a tutorial: https://github.com/TinkerTools/tinker-hp/blob/master/GPU/Deep-HP.md.

## Author contributions

T. J. I., O. A. and T. P. performed simulations; O. A., O. I., T. J. I., L. L. and T. P. contributed the new code; L. L., O. I., T. J. I., P. R., T. P., and J.-P. P. contributed the new methodology; T. J. I., L. L., P. R., and J.-P. P. contributed the analytical tool; T. J. I., L. L., O. I., P. R., H. G., and J.-P. P. analyzed the data. T. J. I., L. L., T. P., H. G., O. I. and J.-P. P. wrote the paper; J.-P. P. designed the research.

## Conflicts of interest

There are no conflicts to declare.

## Supplementary Material

SC-014-D2SC04815A-s001

## References

[cit1] Jumper J., Evans R., Pritzel A., Green T., Figurnov M., Ronneberger O., Tunyasuvunakool K., Bates R., Žídek A., Potapenko A., Bridgland A., Meyer C., Kohl S. A. A., Ballard A. J., Cowie A., Romera-Paredes B., Nikolov S., Jain R., Adler J., Back T., Petersen S., Reiman D., Clancy E., Zielinski M., Steinegger M., Pacholska M., Berghammer T., Bodenstein S., Silver D., Vinyals O., Senior A. W., Kavukcuoglu K., Kohli P., Hassabis D. (2021). Nature.

[cit2] Jaffrelot Inizan T., Célerse F., Adjoua O., El Ahdab D., Jolly L.-H., Liu C., Ren P., Montes M., Lagarde N., Lagardère L., Monmarché P., Piquemal J.-P. (2021). Chem. Sci..

[cit3] El Ahdab D., Lagardère L., Inizan T. J., Célerse F., Liu C., Adjoua O., Jolly L.-H., Gresh N., Hobaika Z., Ren P., Maroun R. G., Piquemal J.-P. (2021). J. Phys. Chem. Lett..

[cit4] El Khoury L., Jing Z., Cuzzolin A., Deplano A., Loco D., Sattarov B., Hédin F., Wendeborn S., Ho C., El Ahdab D., Jaffrelot Inizan T., Sturlese M., Sosic A., Volpiana M., Lugato A., Barone M., Gatto B., Macchia M. L., Bellanda M., Battistutta R., Salata C., Kondratov I., Iminov R., Khairulin A., Mykhalonok Y., Pochepko A., Chashka-Ratushnyi V., Kos I., Moro S., Montes M., Ren P., Ponder J. W., Lagardère L., Piquemal J.-P., Sabbadin D. (2022). Chem. Sci..

[cit5] Phillips J. C., Hardy D. J., Maia J. D. C., Stone J. E., Ribeiro J. V., Bernardi R. C., Buch R., Fiorin G., Hénin J., Jiang W., McGreevy R., Melo M. C. R., Radak B. K., Skeel R. D., Singharoy A., Wang Y., Roux B., Aksimentiev A., Luthey-Schulten Z., Kalé L. V., Schulten K., Chipot C., Tajkhorshid E. (2020). J. Chem. Phys..

[cit6] Shaw D. E. , Grossman J. P., Bank J. A., Batson B., Butts J. A., Chao J. C., Deneroff M. M., Dror R. O., Even A., Fenton C. H., Forte A., Gagliardo J., Gill G., Greskamp B., Ho C. R., Ierardi D. J., Iserovich L., Kuskin J. S., Larson R. H., Layman T., Lee L., Lerer A. K., Li C., Killebrew D., Mackenzie K. M., Mok S. Y., Moraes M. A., Mueller R., Nociolo L. J., Peticolas J. L., Quan T., Ramot D., Salmon J. K., Scarpazza D. P., Schafer U. B., Siddique N., Snyder C. W., Spengler J., Tang P. T. P., Theobald M., Toma H., Towles B., Vitale B., Wang S. C. and Young C., SC ’14: Proceedings of the International Conference for High Performance Computing, Networking, Storage and Analysis, 2014, pp. 41–53

[cit7] Abraham M. J., Murtola T., Schulz R., Páll S., Smith J. C., Hess B., Lindahl E. (2015). SoftwareX.

[cit8] Kobayashi C., Jung J., Matsunaga Y., Mori T., Ando T., Tamura K., Kamiya M., Sugita Y. (2017). J. Comput. Chem..

[cit9] Ponder J. W. and Case D. A., Protein Simulations, Academic Press, 2003, vol. 66, pp. 27–85 10.1016/s0065-3233(03)66002-x 14631816

[cit10] Monticelli L. and Tieleman D. P., in Force Fields for Classical Molecular Dynamics, ed. L. Monticelli and E. Salonen, Humana Press, Totowa, NJ, 2013, pp. 197–213 10.1007/978-1-62703-017-5_8 23034750

[cit11] Gresh N., Cisneros G. A., Darden T. A., Piquemal J.-P. (2007). J. Chem. Theory Comput..

[cit12] Melcr J., Piquemal J.-P. (2019). Front. Mol. Biosci..

[cit13] Shi Y. , Ren P., Schnieders M. and Piquemal J.-P., in Polarizable Force Fields for Biomolecular Modeling, John Wiley and Sons, Ltd, 2015, ch. 2, pp. 51–86

[cit14] Jing Z., Liu C., Cheng S. Y., Qi R., Walker B. D., Piquemal J.-P., Ren P. (2019). Annu. Rev. Biophys..

[cit15] Lagardère L., Jolly L.-H., Lipparini F., Aviat F., Stamm B., Jing Z. F., Harger M., Torabifard H., Cisneros G. A., Schnieders M. J., Gresh N., Maday Y., Ren P. Y., Ponder J. W., Piquemal J.-P. (2018). Chem. Sci..

[cit16] Huang J., Lopes P. E. M., Roux B., MacKerell A. D. (2014). J. Phys. Chem. Lett..

[cit17] Adjoua O., Lagardère L., Jolly L.-H., Durocher A., Very T., Dupays I., Wang Z., Inizan T. J., Célerse F., Ren P., Ponder J. W., Piquemal J.-P. (2021). J. Chem. Theory Comput..

[cit18] Bartók A. P., Payne M. C., Kondor R., Csányi G. (2010). Phys. Rev. Lett..

[cit19] Thompson A., Swiler L., Trott C., Foiles S., Tucker G. (2015). J. Comput. Phys..

[cit20] Vovk V. , in Kernel Ridge Regression, ed. B. Schölkopf, Z. Luo and V. Vovk, Springer, Berlin, Heidelberg, 2013, pp. 105–116

[cit21] Sauceda H. E., Chmiela S., Poltavsky I., Müller K.-R., Tkatchenko A. (2019). J. Chem. Phys..

[cit22] Chmiela S., Tkatchenko A., Sauceda H. E., Poltavsky I., Schütt K. T., Müller K.-R. (2017). Sci. Adv..

[cit23] Chmiela S., Sauceda H. E., Müller K.-R., Tkatchenko A. (2018). Nat. Commun..

[cit24] Sauceda H. E., Chmiela S., Poltavsky I., Müller K.-R., Tkatchenko A. (2019). J. Chem. Phys..

[cit25] Ivanciuc O. , in Applications of Support Vector Machines in Chemistry, John Wiley and Sons, Ltd, 2007, ch. 6, pp. 291–400

[cit26] Bartók A. P., Kondor R., Csányi G. (2013). Phys. Rev. B.

[cit27] Fabrizio A., Briling K. R., Corminboeuf C. (2022). Digital Discovery.

[cit28] Behler J., Parrinello M. (2007). Phys. Rev. Lett..

[cit29] Gastegger M., Schwiedrzik L., Bittermann M., Berzsenyi F., Marquetand P. (2018). J. Chem. Phys..

[cit30] Behler J. (2021). Chem. Rev..

[cit31] Smith J. S., Isayev O., Roitberg A. E. (2017). Chem. Sci..

[cit32] Lier B., Poliak P., Marquetand P., Westermayr J., Oostenbrink C. (2022). J. Phys. Chem. Lett..

[cit33] Jia W. , Wang H., Chen M., Lu D., Lin L., Car R., Weinan E. and Zhang L., SC20: International conference for high performance computing, networking, storage and analysis, 2020, pp. 1–14

[cit34] Ko T. W., Finkler J. A., Goedecker S., Behler J. (2021). Nat. Commun..

[cit35] Loco D., Lagardère L., Caprasecca S., Lipparini F., Mennucci B., Piquemal J.-P. (2017). J. Chem. Theory Comput..

[cit36] Loco D., Lagardère L., Adjoua O., Piquemal J.-P. (2021). Acc. Chem. Res..

[cit37] Loco D., Lagardère L., Cisneros G. A., Scalmani G., Frisch M., Lipparini F., Mennucci B., Piquemal J.-P. (2019). Chem. Sci..

[cit38] Ren P., Ponder J. W. (2003). J. Phys. Chem. B.

[cit39] Ponder J. W., Wu C., Ren P., Pande V. S., Chodera J. D., Schnieders M. J., Haque I., Mobley D. L., Lambrecht D. S., DiStasio R. A., Head-Gordon M., Clark G. N. I., Johnson M. E., Head-Gordon T. (2010). J. Phys. Chem. B.

[cit40] Allinger N. L., Yuh Y. H., Lii J. H. (1989). J. Am. Chem. Soc..

[cit41] Thole B. T. (1981). Chem. Phys..

[cit42] Halgren T. A. (1992). J. Am. Chem. Soc..

[cit43] Walker B., Liu C., Wait E., Ren P. (2022). J. Comput. Chem..

[cit44] Essmann U., Perera L., Berkowitz M. L., Darden T., Lee H., Pedersen L. G. (1995). J. Chem. Phys..

[cit45] Lagardère L., Lipparini F., Polack E., Stamm B., Cances E., Schnieders M., Ren P., Maday Y., Piquemal J.-P. (2015). J. Chem. Theory Comput..

[cit46] Ponder J. W., Wu C., Ren P., Pande V. S., Chodera J. D., Schnieders M. J., Haque I., Mobley D. L., Lambrecht D. S., DiStasio Jr R. A. (2010). *et al.*. J. Phys. Chem. B.

[cit47] Grossfield A., Ren P., Ponder J. W. (2003). J. Am. Chem. Soc..

[cit48] Wu J. C., Piquemal J.-P., Chaudret R., Reinhardt P., Ren P. (2010). J. Chem. Theory Comput..

[cit49] Shi Y., Xia Z., Zhang J., Best R., Wu C., Ponder J. W., Ren P. (2013). J. Chem. Theory Comput..

[cit50] Zhang C., Lu C., Jing Z., Wu C., Piquemal J.-P., Ponder J. W., Ren P. (2018). J. Chem. Theory Comput..

[cit51] Smith J. S., Nebgen B., Lubbers N., Isayev O., Roitberg A. E. (2018). J. Chem. Phys..

[cit52] Smith J. S., Isayev O., Roitberg A. E. (2017). Sci. Data.

[cit53] Smith J. S., Zubatyuk R., Nebgen B., Lubbers N., Barros K., Roitberg A. E., Isayev O., Tretiak S. (2020). Sci. Data.

[cit54] Devereux C., Smith J. S., Huddleston K. K., Barros K., Zubatyuk R., Isayev O., Roitberg A. E. (2020). J. Chem. Theory Comput..

[cit55] Zhang L., Han J., Wang H., Car R., E W. (2018). Phys. Rev. Lett..

[cit56] Wang H., Zhang L., Han J., E W. (2018). Comput. Phys. Commun..

[cit57] Lahey S.-L. J., Rowley C. N. (2020). Chem. Sci..

[cit58] Norberg J., Nilsson L. (2000). Biophys. J..

[cit59] Paszke A. , Gross S., Massa F., Lerer A., Bradbury J., Chanan G., Killeen T., Lin Z., Gimelshein N., Antiga L., Desmaison A., Kopf A., Yang E., DeVito Z., Raison M., Tejani A., Chilamkurthy S., Steiner B., Fang L., Bai J. and Chintala S., Advances in Neural Information Processing Systems, Curran Associates, Inc., 2019, vol. 32, pp. 8024–8035

[cit60] Abadi M. , Agarwal A., Barham P., Brevdo E., Chen Z., Citro C., Corrado G. S., Davis A., Dean J., Devin M., Ghemawat S., Goodfellow I., Harp A., Irving G., Isard M., Jia Y., Jozefowicz R., Kaiser L., Kudlur M., Levenberg J., Mané D., Monga R., Moore S., Murray D., Olah C., Schuster M., Shlens J., Steiner B., Sutskever I., Talwar K., Tucker P., Vanhoucke V., Vasudevan V., Viégas F., Vinyals O., Warden P., Wattenberg M., Wicke M., Yu Y. and Zheng X., TensorFlow: Large-Scale Machine Learning on Heterogeneous Systems, 2015, https://www.tensorflow.org/

[cit61] Chollet F. , *et al.*, Keras, 2015, https://github.com/fchollet/keras

[cit62] MacKerell Jr A. D. , Brooks B., Brooks III C. L., Nilsson L., Roux B., Won Y. and Karplus M., in CHARMM: The Energy Function and Its Parameterization, Wiley and Sons, 2002

[cit63] Tuckerman M., Berne B., Martyna G. (1992). J. Chem. Phys..

[cit64] Lagardère L., Aviat F., Piquemal J.-P. (2019). J. Phys. Chem. Lett..

[cit65] Leimkuhler B., Matthews C. (2013). J. Chem. Phys..

[cit66] Zhou R., Harder E., Xu H., Berne B. J. (2001). J. Chem. Phys..

[cit67] Liberatore E., Meli R., Rothlisberger U. (2018). J. Chem. Theory Comput..

[cit68] Bussi G., Donadio D., Parrinello M. (2007). J. Chem. Phys..

[cit69] Bennett C. H. (1976). J. Comput. Phys..

[cit70] Hénin J. , Lelièvre T., Shirts M. R., Valsson O. and Delemotte L., Enhanced sampling methods for molecular dynamics simulations, 2022

[cit71] Zhang J., Shi Y., Ren P. (2012). Protein-Ligand Interact..

[cit72] Mohamed N. A., Bradshaw R. T., Essex J. W. (2016). J. Comput. Chem..

[cit73] Wu J. C., Chattree G., Ren P. (2012). Theor. Chem. Acc..

[cit74] Ghahremanpour M. M., Tirado-Rives J., Deshmukh M., Ippolito J. A., Zhang C.-H., Cabeza de Vaca I., Liosi M.-E., Anderson K. S., Jorgensen W. L. (2020). ACS Med. Chem. Lett..

[cit75] Laury M. L., Wang Z., Gordon A. S., Ponder J. W. (2018). J. Comput.-Aided Mol. Des..

[cit76] Shi Y., Laury M. L., Wang Z., Ponder J. W. (2021). J. Comput.-Aided Mol. Des..

[cit77] Amezcua M., El Khoury L., Mobley D. L. (2021). J. Comput.-Aided Mol. Des..

[cit78] Bell D. R., Qi R., Jing Z., Xiang J. Y., Mejias C., Schnieders M. J., Ponder J. W., Ren P. (2016). Phys. Chem. Chem. Phys..

[cit79] Amezcua M., Setiadi J., Ge Y., Mobley D. L. (2022). J. Comput.-Aided Mol. Des..

[cit80] Harger M., Li D., Wang Z., Dalby K., Lagardère L., Piquemal J.-P., Ponder J., Ren P. (2017). J. Comput. Chem..

[cit81] Muddana H. S., Fenley A. T., Mobley D. L., Gilson M. K. (2014). J. Comput.-Aided Mol. Des..

[cit82] Jaffrelot Inizan T., Célerse F., Adjoua O., El Ahdab D., Jolly L.-H., Liu C., Ren P., Montes M., Lagarde N., Lagardère L., Monmarché P., Piquemal J.-P. (2021). Chem. Sci..

[cit83] Monmarché P., Weisman J., Lagardère L., Piquemal J.-P. (2020). J. Chem. Phys..

[cit84] Zhang L., Wang H., Muniz M. C., Panagiotopoulos A. Z., Car R., W. E. (2022). J. Chem. Phys..

[cit85] Tu N. T. P., Rezajooei N., Johnson E. R., Rowley C. (2023). Digital Discovery.

[cit86] Plé T. , Lagardère L. and Piquemal J.-P., Force-Field-Enhanced Neural Network Interactions: from Local Equivariant Embedding to Atom-in-Molecule properties and long-range effects, 2023, https://arxiv.org/abs/2301.08734 10.1039/d3sc02581k PMC10646944 38020379

[cit87] Poier P. P., Lagardère L., Piquemal J.-P. (2022). J. Chem. Theory Comput..

[cit88] Poier P. P., Jaffrelot Inizan T., Adjoua O., Lagardère L., Piquemal J.-P. (2022). J. Phys. Chem. Lett..

