# Supplementary material for: Scalable hybrid deep neural networks/polarizable potentials biomolecular simulations including long-range effects
Source: Chem Sci. 2023 Apr 4;14(20):5438–52. doi: 10.1039/d2sc04815a (PMC10208042; doi:10.1039/d2sc04815a)
Supplement: SC-014-D2SC04815A-s001 [file SC-014-D2SC04815A-s001.pdf]

Electronic Supplementary Material (ESI) for Chemical Science.
This journal is © The Royal Society of Chemistry 2023

Scalable Hybrid Deep Neural Networks/Polarizable Potentials Biomolecular Simulations including Long-Range Effects

Théo Jaffrelot Inizan,[†] Thomas Plé,[†] Olivier Adjoua,[†] Pengyu Ren,[‡] Hatice Gökcan,[¶] Olexandr Isayev,[¶] Louis Lagardère,[†,§] and Jean-Philip Piquemal[*,†,‡,||]

†*Sorbonne Université, LCT, UMR 7616 CNRS, Paris, France*

‡*The University of Texas at Austin, Department of Biomedical Engineering, TX, USA*

¶*Carnegie Mellon University, Department of Chemistry, Pittsburgh, Pennsylvania, USA*

§*Sorbonne Université, IP2CT, FR 2622 CNRS, Paris, France*

||*Institut Universitaire de France, Paris, France*

E-mail: jean-philip.piquemal@sorbonne-universite.fr

1 Study of the Deep-HP Platform Scalability

a) b)

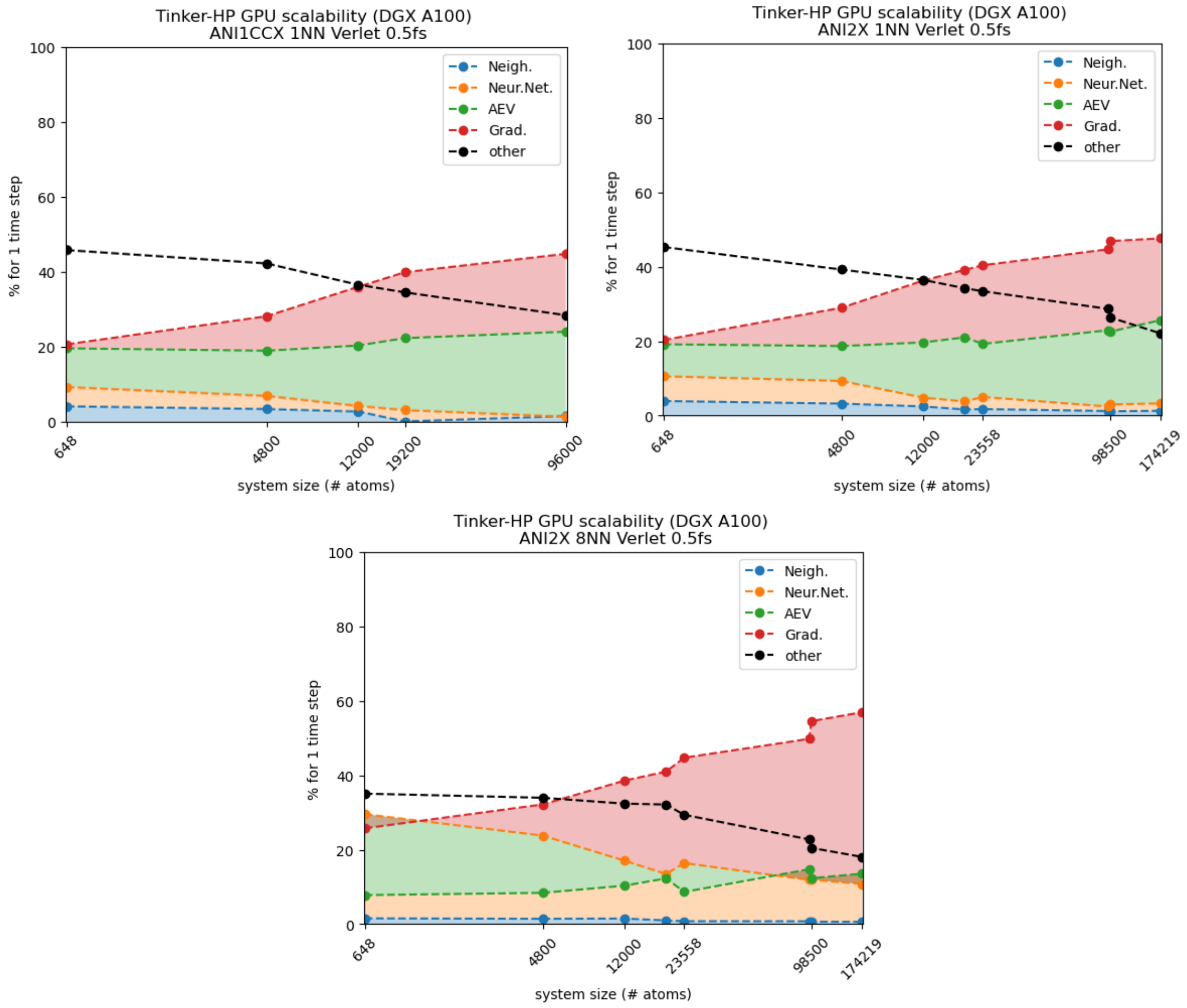

Figure S1: Performance plot comparison between ANI-2x(1NN), ANI-2x(8NN) and ANI-1ccx(1NN) for increasing system size on 1 GPU A100 in the NVE ensemble and using Verlet 0.5 fs time-step.

2 Hybrid Polarizable/ML potential model

2.1 Multi-timestep integrators: RESPA and RESPA1

Table S1. Relative performances of usual AMOEBA setups: Velocity Verlet 0.2fs, Velocity Verlet 1fs and BAOAB-RESPA (0.25/2fs) compared to ANI alone (denoted ANI) and the hybrid ANI-2X/AMOEBA approach (denoted A/A) using several simulation setups for the solvated benzene system (3000 atoms).

	ANI(0.2)	A/A(0.2)	A/A(0.25/1)	A/A(0.25/2)	A/A(0.25/2/4)	A/A(0.25/2/6)
AMOEBA V 0.2fs	10.78	8.62	1.81	1.02	0.61	0.48
AMOEBA V 1fs	44.64	35.69	7.52	4.24	2.53	1.96
AMOEBA R 0.25/2	49.50	39.58	8.35	4.70	2.80	2.18
Integrator-type	V	V	R	R	R1	$R1^H$

Table S2. Production speed in ns per day of AMOEBA and ANI-2x with Velocity-Verlet (V) integrator using 0.2fs and 0.5fs time-step.

A100	waterhuge (12000)	cox (174219)
ANI-2x V 0.2fs	1.02	0.084
ANI-2x V 0.5fs	2.55	0.21
AMOEBA V 0.2fs	6.54	0.90
AMOEBA V 0.5fs	14.18	1.96
Ratio V 0.2fs	**6.41**	**10.71**
Ratio V 0.5fs	**5.56**	**9.33**

2.2 ANI-2X/AMOEBA Binding Free Energies through AMOEBA simulation

In this section, we explain the basic principles of the reweighted Benett Acceptance Ratio (BAR) method. Let's denote by i and $i+1$, two states for which we want to compute the free energy difference $\Delta F'_{i,i+1} = F'_{i+1} - F'_i = -\frac{1}{\beta}\ln\langle e^{-\beta(U'_{i+1}-U'_i)}\rangle_i$ characterized by reduced energies U_i and U_{i+1}. Common estimators such as BAR is based on the assumption of overlap meaning each configurations have non-negligible probability under the two states. For example, when performing alchemical free energies, the two states have different decoupling/scaling factor on the Van der Waals and electrostatic potentials. $\Delta F'_{i,i+1}$ can be estimate by using the energy differences, $\Delta U'_{i+1,i}$ sampled in state $i+1$, $\Delta U'_{i,i+1}$ sampled in state i, and the BAR estimator, the latter being constructed to minimize the variance and the mean-squared error. Within the targeted ensemble U', the BAR estimator consists of solving self-consistently, over k:

$$\begin{aligned}\Delta F'_{i,i+1}(k+1) &= -\frac{1}{\beta}\ln\frac{\langle 1/[1+\exp\beta(U'_i - U'_{i+1} + C)]\rangle'_{i+1}}{\langle 1/[1+\exp\beta(U'_{i+1} - U'_i + C)]\rangle'_i} + C \\ &= -\frac{1}{\beta}\ln\frac{\frac{\int d^N\mathbf{r}(1/[1+\exp\beta(U'_i-U'_{i+1}+C)])e^{-\beta(U'_{i+1})}}{\int d^N\mathbf{r}e^{-\beta(U'_{i+1})}}}{\frac{\int d^N\mathbf{r}(1/[1+\exp\beta(U'_{i+1}-U'_i+C)])e^{-\beta(U'_i)}}{\int d^N\mathbf{r}e^{-\beta(U'_i)}}} + C\end{aligned} \quad (1)$$

with $C = \Delta F'_{i,i+1}(k)$ and β the thermodynamic beta.

Now, let's suppose we have two ensemble U and U' with small overlap for each states i. If we want to obtain $\Delta F'_{i,i+1}$ from this other ensemble U, we can notice:

$$\langle 1/[1+\exp\beta(U'_i - U'_{i+1} + C)]\rangle'_{i+1} = \frac{\int d^N\mathbf{r}(1/[1+\exp{(U'_i - U'_{i+1} + C)/RT}])e^{-\beta(U'_{i+1}-U_{i+1})}e^{-\beta(U_{i+1})}}{\int d^N\mathbf{r}e^{-\beta(U'_{i+1}-U_{i+1})}e^{-\beta(U_{i+1})}}$$
$$= \frac{\int d^N\mathbf{r}(1/[1+\exp{(U'_i - U'_{i+1} + C)/RT}])e^{-\beta(U'_{i+1}-U_{i+1})}e^{-\beta(U_{i+1})}}{\int d^N\mathbf{r}e^{-\beta(U_{i+1})}}$$
$$X\frac{\int d^N\mathbf{r}e^{-\beta(U_{i+1})}}{\int d^N\mathbf{r}e^{-\beta(U'_{i+1}-U_{i+1})}e^{-\beta(U_{i+1})}}$$
$$= \frac{\langle[\exp\beta(U'_{i+1} - U_{i+1})]/[1+\exp\beta(U'_i - U'_{i+1} + C)]\rangle_{i+1}}{\langle\exp\beta(U'_{i+1} - U_{i+1})\rangle_{i+1}} \tag{2}$$

With Eq. (1), this gives the reweighted BAR formula, Eq. (3):

$$\Delta F(k+1) = \frac{\langle[\exp\beta(U'_{i+1} - U_{i+1})]/[1+\exp\beta(U'_i - U'_{i+1} + C)]\rangle_{i+1}/\langle\exp\beta(U'_{i+1} - U_{i+1})\rangle_{i+1}}{\langle[\exp\beta(U'_i - U_i)]/[1+\exp\beta(U'_{i+1} - U'_i + C)]\rangle_{i+1}/\langle\exp\beta(U'_i - U_i)\rangle_i} \tag{3}$$

Because it has been shown that AMOEBA and ANI-2X/AMOEBA share an overlap with numerous ligand, in the following we used U' as a target hybrid ANI-2X/AMOEBA potential and U as the AMOEBA reference. In addition to aleviate the intrinsic sampling problem of machine learning potential it allows to estimate the free energy of systems which may not be stable with the MLP during a dynamic, such as in our case the C2/C3/C4 host-guest complexes.

2.3 Solvation free energies

In the following, the experimental solvation free energies are taken from the Minnesota solvation database and from Abraham et al. The AMOEBA solvation free energies of Table S1-1, S1-2 were taken from Ren et al. (Poltype 2) and from Essex et al. for Table S2, S3, s4.

Table S3. ANI-2X/AMOEBA calculated solvation free energies for small molecules in water (ϵ=78.30) against AMOEBA and experimental data. The lowest absolute error is in green. ^{R} have been re-parametrized with a newer version of Poltype 2. ANI-2X/AMOEBA RMSE: 0.78 kcal/mol; **22**/38. AMOEBA RMSE: 0.68 kcal/mol; **17**/38.

		$\Delta G_{solv}(kcal/mol)$		$\Delta\Delta G_{solv}(kcal/mol)$	
Molecule	Exp	ANI/AMOEBA	AMOEBA	ANI/AMOEBA	AMOEBA
Methylsulfide	-1.24	-0.87(-0.80)	-0.83	**0.37**(0.44)	0.41
Methylethylsulfide	-1.50	-1.38(-1.33)	-1.28	**0.12**(0.17)	0.22
Water^R	-6.32	-6.29(-6.23)	-5.79	**0.03**(0.09)	0.53
Trimethylamine	-3.24	-2.28(-2.16)	-2.36	0.96(1.08)	**0.88**
Toluene	-0.89	-0.35(-0.36)	-0.26	0.44(**0.43**)	0.63
Propanol	-4.83	-5.20(-5.17)	-5.30	0.37(**0.34**)	0.47
Propane	1.96	2.10(2.10)	2.06	0.14(0.14)	**0.10**
Phenol^R	-6.62	-5.38(-5.45)	-5.25	1.24(1.17)	**1.14**
p-cresol	-6.14	-6.12(-6.13)	-6.27	0.02(**0.01**)	0.13
n-butane	2.08	2.20(2.01)	2.23	0.12(**0.07**)	0.15
Methylether	-1.90	-1.13(-1.26)	-1.22	0.77(**0.64**)	0.68
Methylamine^R	-4.56	-5.39(-5.39)	-5.45	0.83(**0.83**)	0.89
Methanol	-5.11	-5.10(-5.08)	-5.14	**0.01**(0.03)	0.03
Methane	1.99	1.68(1.68)	1.73	0.31(0.31)	**0.27**
Isopropanol	-4.76	-6.11(-6.01)	-5.99	1.35(1.25)	**1.23**
Imidazole	-9.63	-10.12(-10.08)	-10.40	0.49(**0.45**)	0.77
Hydrogensulfide	-0.44	-0.06(-0.08)	-0.68	0.38(0.36)	**0.24**
Ethylbenzene	-0.80	-0.57(-0.48)	-0.16	**0.23**(0.32)	0.64
Ethylamine	-4.50	-3.79(-3.93)	-4.08	0.71(0.57)	**0.42**
Ethanol	-5.00	-5.38(-5.19)	-5.39	0.38(**0.19**)	0.39
Ethane	1.83	1.98(1.93)	1.82	0.15(0.10)	**0.01**
Dimethylsulfide	-1.54	-1.20(-1.26)	-1.25	0.34(**0.28**)	0.29
Dimethylamine^R	-4.29	-2.25(-2.29)	-2.41	2.04(2.00)	**1.88**
Diethylsulfide	-1.60	-1.10(-1.38)	-1.33	0.50(**0.12**)	0.27
Benzene	-0.87	-0.22(-0.52)	-0.37	0.65(**0.35**)	0.50

Table S4. ANI-2X/AMOEBA calculated solvation free energies for small molecules in water (ϵ=78.30) against AMOEBA and experimental data. The lowest absolute error are in green. ANI-2X/AMOEBA RMSE: 0.78 kcal/mol; **22**/38. AMOEBA RMSE: 0.68 kcal/mol; **17**/38.

		$\Delta G_{solv}(kcal/mol)$		$\Delta\Delta G_{solv}(kcal/mol)$	
Molecule	Exp	ANI/AMOEBA	AMOEBA	ANI/AMOEBA	AMOEBA
Aceticacid	-6.70	-4.31(-4.24)	-4.95	2.39(2.46)	**1.75**
Formicacid	-5.11	-4.84(-4.96)	-4.98	0.27(0.15)	**0.13**
Propylamine	-4.40	-4.31(-4.42)	-4.63	0.09(**0.02**)	0.23
Dimethyldisulfide	-1.83	-0.49(-0.31)	-1.11	1.34(1.52)	**0.72**
Methylisopropylether	-2.01	-2.70(-2.52)	-2.15	0.69(0.51)	**0.14**
Di-n-propylether	-1.16	-1.65(-1.32)	-1.33	0.49(**0.16**)	0.17
Methanethiol	-1.24	-0.77(-0.83)	-0.83	0.47(**0.41**)	**0.41**
Octan-1-ol	-4.09	-5.36(-5.57)	-4.73	1.27(1.48)	**0.64**
n-octane	2.88	2.88(2.81)	3.44	**0.00**(0.07)	0.56
Di-n-propylsulfide	-1.28	-2.04(-1.71)	-0.53	0.76(**0.43**)	0.75
Methylacetate	-3.13	-2.41(-2.53)	-2.89	0.72(0.60)	**0.24**
22-Dimethylbutane	2.51	2.37(2.30)	2.85	**0.14**(0.21)	0.34
n-butanethiol	-0.99	-0.19(-0.34)	-0.48	0.80(0.65)	**0.51**

Table S5. ANI-2X/AMOEBA calculated solvation free energies for small molecules in toluene (ϵ=2.38) against AMOEBA and experimental data. The lowest absolute error are in green. ANI-2X/AMOEBA RMSE: 0.93 kcal/mol ± 0.03 (BAR error calculated via boostraping); **10**/20. AMOEBA RMSE: 1.06 kcal/mol; **9**/20.

		$\Delta G_{solv}(kcal/mol)$		$\Delta\Delta G_{solv}(kcal/mol)$	
Molecule	Exp	ANI/AMOEBA	AMOEBA	ANI/AMOEBA	AMOEBA
1,4-dioxane	-4.91	-5.48	-5.43	0.57	**0.52**
2-butanone	-4.27	-3.82	-3.74	**0.45**	0.53
Acetic acid	-4.00	-3.28	-3.24	**0.72**	0.76
Acetone	-3.59	-4.14	-4.09	0.55	**0.50**
Ammonia	-2.38	-0.45	0.42	**1.93**	2.80
Aniline	-6.69	-5.64	-5.11	**1.05**	1.58
Ethanol	-3.33	-2.70	-2.60	**0.63**	0.73
Methanol	-2.18	-2.05	-2.10	0.13	**0.08**
Methylamine	-2.65	-2.01	-2.88	0.64	**0.23**
n-octane	-5.38	-4.13	-3.82	**1.25**	1.56
Nitromethane	-4.31	-3.90	-4.00	0.41	**0.31**
Phenol	-6.93	-5.63	-5.33	**1.30**	1.60
Pyridine	-5.13	-4.74	-4.58	**0.39**	0.55
Toluene	-5.12	-3.97	-4.04	1.15	**1.08**
Diethylamine	-3.75	-3.23	-2.58	**0.52**	1.17
Trimethylamine	-2.71	-3.27	-3.33	**0.56**	0.62
Hexanoic acid	-6.97	-5.53	-5.83	1.44	**1.14**
Methylacetate	-3.81	-3.74	-3.72	**0.07**	0.09
Methylbenzoate	-7.96	-6.53	-7.18	1.43	**0.78**
Hydrogen peroxide	-3.14	-2.31	-3.34	0.83	**0.20**

Table S6. ANI-2X/AMOEBA calculated solvation free energies, with BAR error, for small molecules in acetonitrile (ϵ=36.64) against AMOEBA and experimental data. The lowest absolute error are in green. ANI-2X/AMOEBA RMSE: 0.69 kcal/mol ± 0.02 (BAR error calculated via boostraping); **4**/6. AMOEBA RMSE: 0.71 kcal/mol ± 0.03; **2**/6.

		$\Delta G_{solv}(kcal/mol)$		$\Delta\Delta G_{solv}(kcal/mol)$	
Molecule	Exp	ANI/AMOEBA	AMOEBA	ANI/AMOEBA	AMOEBA
1,4-dioxane	-5.33	-5.64	-5.55	0.31	**0.22**
2-butanone	-4.73	-3.74	-3.78	0.99	**0.95**
Ethanol	-4.43	-3.74	-3.66	**0.69**	0.77
n-octane	-3.57	-3.71	-3.86	**0.14**	0.29
Nitromethane	-5.62	-4.66	-4.64	**0.96**	0.98
Toluene	-4.68	-4.07	-4.04	**0.61**	0.64

Table S7. ANI-2X/AMOEBA calculated solvation free energies, with BAR error, for small molecules in DMSO (ϵ=47.24) against AMOEBA and experimental data. The lowest absolute error are in green. ANI-2X/AMOEBA RMSE: 0.80 kcal/mol ± 0.03; **4**/6. AMOEBA RMSE: 1.21 kcal/mol ± 0.03 (BAR error calculated via boostraping); **2**/6.

		$\Delta G_{solv}(kcal/mol)$		$\Delta\Delta G_{solv}(kcal/mol)$	
Molecule	Exp	ANI/AMOEBA	AMOEBA	ANI/AMOEBA	AMOEBA
1,4-dioxane	-4.90	-5.40	-5.27	0.50	**0.37**
2-butanone	-4.23	-3.05	-2.87	**0.96**	1.36
Ethanol	-5.25	-4.59	-4.48	**0.66**	0.77
n-octane	-2.84	-2.80	-1.04	**0.04**	1.80
Nitromethane	-5.66	-4.45	-4.56	1.21	**1.10**
Toluene	-4.42	-3.85	-3.09	**1.03**	1.33

Table S8. ANI-2X/AMOEBA (A/A) absolute binding free energies for the G9 guest of the SAMPL6 challenge against AMOEBA and experimental data. Simulations performed in the NPT ensemble using an RESPA (0.2/2) integrator with 2ns BAR windows.
$\Delta G_{bind} = \Delta G_{host-guest} - \Delta G_{solvation} + G_{correction}$ with $G_{correction} = 1.7$ kcal/mol
Experimental value: ΔG_{exp} = -8.68 kcal/mol.

Model	ΔG_{bulk}	$\Delta G_{host-guest}$	ΔG_{bind}	$\Delta\Delta G$
ANI/AMOEBA RESPA 2fs[a]	52.57	-62.96	-8.69	**0.01**
AMOEBA ref.	3.27	-13.79	-8.82	0.14

Table S9. ANI-2X/AMOEBA absolute binding free energies for complexes of the SAMPL4 challenge against AMOEBA and experimental data. Simulations performed in the NPT ensemble using an RESPA (0.2/2) integrator with 2ns and 5ns (in parentheses) BAR windows. * (C10) uses RESPA (0.25/1). ** (C2, C3, C4) value obtained using an ANI-2X/AMOEBA reweighting of classical AMOEBA trajectories with 5ns BAR windows. For C3 we also performed 10 ns BAR windows (in parenthese).

$\Delta G_{bind} = \Delta G_{host-guest} - \Delta G_{solvation} + G_{correction}$ with $G_{correction} = 6.245$ kcal/mol

ANI-2X/AMOEBA RMSE: 0.94 kcal/mol ± 0.05 (BAR error calculated via boostraping); **11**/13. AMOEBA RMSE: 1.81 kcal/mol; **2**/13.

			$\Delta G_{solv}(kcal/mol)$		$\Delta\Delta G_{solv}(kcal/mol)$	
Guest	Charge	Exp	ANI/AMOEBA	AMOEBA	ANI/AMOEBA	AMOEBA
C1	2	-9.90	-9.35(-9.96)	-12.27	0.55(**0.06**)	2.37
C2**	1	-9.60	-9.26	-6.46	**0.34**	3.14
C3**	1	-6.60	-4.84(-5.21)	-6.59	1.76	**0.01**
C4**	2	-8.40	-8.47	-11.34	**0.07**	2.94
C6	1	-7.90	-7.33(-7.31)	-6.18	**0.57**(0.59)	1.72
C7	1	-10.10	-9.04(-9.75)	-10.49	1.06(**0.35**)	0.39
C8	1	-11.80	-10.77(-11.10)	-11.84	1.03(0.70)	**0.04**
C9	1	-12.60	-15.93(-15.17)	-15.42	3.33(**2.57**)	2.82
C10*	2	-7.90	-6.44(-6.44)	-5.06	**1.46**(**1.46**)	2.84
C11	1	-11.10	-11.25(-10.86)	-10.48	**0.15**(0.24)	0.62
C12	1	-13.30	-12.29(-13.20)	-12.11	1.01(**0.10**)	1.19
C13	1	-14.10	-14.16(-13.63)	-13.92	**0.06**(0.47)	0.18
C14	1	-11.60	-13.21(-12.19)	-12.41	1.61(**0.59**)	0.81

Table S10. ANI-2X/AMOEBA absolute binding free energies for the C5 complex of the SAMPL4 challenge small molecules against AMOEBA and experimental data. Simulations performed in the NPT ensemble using a RESPA(0.2/2) integrator for ANI-2X/AMOEBA with 2ns or 5ns (in parenthesis) BAR windows.

			$\Delta G_{solv}(kcal/mol)$		$\Delta\Delta G_{solv}(kcal/mol)$	
Guest	Charge	Exp	ANI/AMOEBA	AMOEBA	ANI/AMOEBA	AMOEBA
C5	1	-8.50	-3.37(-2.11)	-3.37	**5.13**(6.39)	**5.13**
C5b	2	-8.50	-3.69(-3.36)	-3.39	**4.81**(5.14)	5.11
C5b_2	2	-8.50	-13.12(-14.66)	-	4.62(6.16)	-

Table S11. ANI-2X/AMOEBA calculated solvation free energies for small molecules in water (ϵ=78.30). Statistical uncertainties assessment with 2 replicas. Averaged statistical uncertainties: 0.17 kcal/mol; Averaged BAR error: 0.04 kcal/mol.

	$\Delta G_{solv}(kcal/mol)$		$\Delta\Delta G_{solv}(kcal/mol)$	
Molecule	Rep. 1	Rep. 2	$\|\Delta\|$	BAR
Methylsulfide	-0.87	-0.81	0.06	0.03
Methylethylsulfide	-1.38	-1.07	0.31	0.04
Water	-6.29	-6.23	0.06	0.02
Trimethylamine	-2.28	-2.27	0.01	0.03
Toluene	-0.35	-0.36	0.01	0.06
Propanol	-5.20	-5.22	0.02	0.03
Propane	2.10	1.85	0.25	0.03
Phenol	-5.38	-5.44	0.06	0.05
p-cresol	-6.12	-5.91	0.21	0.05
n-butane	2.20	2.06	0.14	0.04
Methylether	-1.13	-0.97	0.26	0.03
Methylamine	-5.39	-5.39	0.00	0.02
Methanol	-5.10	-5.08	0.02	0.03
Methane	1.68	1.68	0.00	0.02
Isopropanol	-6.11	-5.88	0.23	0.04
Imidazole	-10.12	-10.34	0.22	0.03
Hydrogensulfide	-0.06	-0.15	0.09	0.02

Table S12. ANI-2X/AMOEBA calculated solvation free energies for small molecules in water (ϵ=78.30). Statistical uncertainties assessment with 2 replicas. Averaged statistical uncertainties: 0.17 kcal/mol; Averaged BAR error: 0.04 kcal/mol.

	$\Delta G_{solv}(kcal/mol)$		$\Delta\Delta G_{solv}(kcal/mol)$	
Molecule	Rep. 1	Rep. 2	$\|\Delta\|$	BAR
Ethylbenzene	-0.57	-0.15	0.42	0.06
Ethylamine	-3.79	-3.84	0.05	0.03
Ethanol	-5.38	-5.28	0.10	0.03
Ethane	1.98	1.82	0.16	0.03
Dimethylsulfide	-1.20	-1.25	0.05	0.04
Dimethylamine	-2.25	-1.95	0.30	0.04
Diethylsulfide	-1.10	-1.70	0.60	0.04
Benzene	-0.22	-0.54	0.32	0.04
Aceticacid	-4.31	-3.99	0.32	0.03
Formicacid	-4.84	-4.71	0.13	0.03
Propylamine	-4.31	-4.15	0.16	0.04
Dimethyldisulfide	-0.49	-0.53	0.04	0.05
Methylisopropylether	-2.70	-2.18	0.52	0.04
Di-n-propylether	-1.65	-1.64	0.01	0.07
Methanethiol	-0.77	-0.63	0.14	0.03
n-octane	2.88	2.86	0.02	0.07
Methylacetate	-2.41	-2.52	0.11	0.04
22-Dimethylbutane	2.37	2.48	0.11	0.05
n-butanethiol	-0.19	-0.56	0.37	0.06
